# A new view of missense mutations in *α*‐mannosidosis using molecular dynamics conformational ensembles

**DOI:** 10.1002/pro.70080

**Published:** 2025-03-24

**Authors:** Špela Mandl, Bruno Di Geronimo, Santiago Alonso‐Gil, Christoph Grininger, Gibu George, Ulrika Ferstl, Sereina Annik Herzog, Bojan Žagrović, Christoph Nusshold, Tea Pavkov‐Keller, Pedro A. Sánchez‐Murcia

**Affiliations:** ^1^ Laboratory of Computer‐Aided Molecular Design, Division of Medicinal Chemistry, Otto‐Loewi Research Center Medical University of Graz Graz Austria; ^2^ Max Perutz Labs Vienna Biocenter Campus (VBC) Vienna Austria; ^3^ Department of Structural and Computational Biology Vienna BioCenter University of Vienna Campus‐Vienna‐Biocenter 5 Vienna Austria; ^4^ Institute of Molecular Biosciences, NAWI Graz University of Graz Graz Austria; ^5^ Institut de Química Computacional i Catàlisi and Departament de Química Universitat de Girona Girona Catalonia Spain; ^6^ Institute for Medical Informatics, Statistics and Documentation Medical University of Graz Graz Austria; ^7^ Field of Excellence BioHealth University of Graz Graz Austria; ^8^ BioTechMed‐Graz Graz Austria; ^9^ Present address: School of Chemistry and Biochemistry Georgia Institute of Technology Atlanta Georgia USA

**Keywords:** alpha‐mannosidosis, bond‐to‐bond propensity, conformational ensemble, free energy, mutual information, rare disease

## Abstract

The mutation of remote positions on enzyme scaffolds and how these residue changes can affect enzyme catalysis is still far from being fully understood. One paradigmatic example is the group of lysosomal storage disorders, where the enzyme activity of a lysosomal enzyme is abolished or severely reduced. In this work, we analyze molecular dynamics simulation conformational ensembles to unveil the molecular features controlling the deleterious effects of the 43 reported missense mutations in the human lysosomal *α*‐mannosidase. Using residue descriptors for protein dynamics, their coupling with the active site, and their impact on protein stability, we have assigned the contribution of each of the missense mutations into protein stability, protein dynamics, and their connectivity with the active site. We demonstrate here that the use of conformational ensembles is a powerful approach not only to better understand missense mutations at the molecular level but also to revisit the missense mutations reported in lysosomal storage disorders in order to aid the treatment of these diseases.

## 1 INTRODUCTION

Lysosomal storage disorders (LSDs) (Filocamo & Morrone, 2011; Moro, 2021; Platt et al., 2018) are a paradigm of how single‐point mutations in an enzyme can disrupt or abolish its activity and cause a pathology (Filocamo & Morrone, 2011; Futerman & Meer, 2004; Moro, 2021; Platt et al., 2018). Looking at the location of these mutations in LSDs‐related enzymes, the reported clinical mutational landscape includes not only positions close to the active site but also remote ones more than 10 Å away from it. Computationally, the effect of the orthosteric mutations can in many cases be assessed using the ‘chemical sense' or by calculating properties of the active site in the presence of the mutation. In contrast, the understanding of how remote mutations allosterically affect the enzyme activity (Bishop et al., 2022) in LSD is still to be understood (Olson et al., 2020). The remote mutation of the side chain of a protein residue in an LSD‐related enzyme can impact its network of amino‐acid interactions in different ways (Lisi & Loria, 2017; Stefl et al., 2013). On the one hand, some of these mutations can prevent the correct folding of the enzyme in the endoplasmic reticulum and/or its transport to the lysosome (Ron & Horowitz, 2005; Wang & Kaufman, 2012). The computation of the change of the free energy of the protein scaffold upon mutation as ΔΔ*G*_*X*→*Y*_ has been shown useful to assess the impact of such pathogenic mutations on the integrity of the enzyme (Gerasimavicius et al., 2020; Gerasimavicius et al., 2022). On the other hand, other mutations may not affect the enzyme folding but reduce significantly its enzymatic activity (Lim et al., 2018). Whereas the former group of mutations affects the folding process of the enzyme, the latter group may introduce changes in the local interactions and/or the dynamics and flexibility of the protein scaffold, causing ‘frustrated networks in proteins' (D'Amico et al., 2020). Recently, the possibility to access a protein structure from its sequence via deep‐learning algorithms has allowed the exploration of these amino‐acid networks from a structural perspective (Alamdari et al., 2023; Jumper et al., 2021; Lin et al., 2023). As an example, there are computational tools available to detect the network of interactions between remote mutations and the active site when the structure is available (Amor et al., 2014; Amor et al., 2016; Mersmann et al., 2021a). The protein structure can also be used as an input file to study the role of enzyme conformational dynamics, an important factor in catalysis (Hammes, 2002; Yabukarski et al., 2022; Yon et al., 1998). Indeed, via classical molecular dynamics (MD) simulations, conformational ensembles of the enzyme with explicit solvation can be obtained (Childers & Daggett, 2018; Platero‐Rochart & Sánchez‐Murcia, 2024). Importantly, by analyzing those conformational ensembles, the role of remote residue on catalysis can be detected (Crean et al., 2023; Fleck et al., 2016; Romero‐Rivera et al., 2017).

Human lysosomal α‐mannosidase (hLAMAN, EC 3.2.1.24) is part of the GH38 family of Zn‐dependent aspartic glycosidase hydrolases (Heikinheimo et al., 2003; Henrissat & Davies, 1997; Park et al., 2005; Rovira et al., 2020). It catalyzes the cleavage of α‐mannosidic linkages (*α*(1→2), *α*(1→3), and *α*(1→6)) from oligosaccharides. Lack of hLAMAN activity causes the autosomal recessive disease termed α‐mannosidosis (MANSA) (Gotoda et al., 1998; Kuokkanen et al., 2011; Malm & Nilssen, 2008; Zielonka et al., 2019). MANSA is an LSD associated with more than 130 pathogenic variants (McCorvie & Yue, 2020; Stensland et al., 2015). The effect of the 43 reported missense mutations in MANSA has been classified depending on their effect on the protein activity or stability (Table 1): 17 of them reduce or abolish the enzymatic activity, 23 yield an incorrect enzyme folding or incomplete trafficking to the lysosome, and 3 of them have not been totally classified yet. As examples, D74E, H200L, or H200N (Riise Stensland et al., 2012; Sbaragli et al., 2005), which lead to alterations in the coordination of metal ions at the active site (Berg et al., 1999), belong to the first group of mutations, whereas R750W, which occurs in over 25% of MANSA patients, disrupts the proper folding of the enzyme (Gotoda et al., 1998). Although the macroscopic effects of these mutations are known, and the malignancy of missense mutations can be predicted by tools like ClinVar (Landrum et al., 2013), the specific molecular mechanisms behind these defective mutations are not yet well understood. Amongst other causes, this is due to the lack of an experimental structural model of hLAMAN. hLAMAN is closely related to bovine lysosomal α‐mannosidase (bLAMAN, EC 3.2.1.24) with an 80% of similarity, for which one crystallographic structure is available (PDB id. 1O7D) (Heikinheimo et al., 2003). bLAMAN is a homodimer, wherein each of the monomers consists of five peptides (labeled as A‐E). Analogously, it is known that hLAMAN is cleaved into three peptides: one of 70 kDa, one of 42 kDa (referred to as D), and one of 13/15 kDa (referred to as E) (Liao et al., 1996). The 70 kDa peptide is subsequently cleaved into three additional peptides (A, B, and C). These A, B, and C peptides are connected by disulfide bonds and exhibit a high degree of glycosylation. Interestingly, although the most similar human Golgi α‐mannosidase II (hAMan II, EC: 3.2.1.114) displays similar kinetic properties, an 81% amino acid similarity, and a similar inhibitor sensitivity/substrate specificity (Elsen, 2001; Foster et al., 1995; Liao et al., 1996), this cytoplasmic enzyme appears as a single monomer (Vallée et al., 2000).

**TABLE 1:** Reported deleterious missense mutations in hLAMAN (Stensland et al., 2015).

Mutant	Chain	Relative activity (%)^a^	Distance (Å)^b^	Secondary structure	Contact^c^
(a) Protein folding					
C55F	A	9	27.3	SS‐bond	B
A95P	A	10	13.0	Loop	‐
P197R	A	12	7.0	Loop	C
R202P	A	24	20.0	α‐helix 8	C,D
L352P	A	11	26.0	β‐sheet 9	‐
T355P	B	9	19.4	α‐helix 13	‐
P356R	B	6	17.9	α‐helix 13	‐
G390C	B	7	17.1	β‐turn	D
H445Y	C	6	8.2	Loop	A
S453Y	C	19	10.2	Loop	A
S453F	C	19	10.2	Loop	A
L565P	C	22	45.1	β‐sheet 20	D
W714R	D	5	32.1	β‐sheet 31	‐
R750W	D	8	24.7	β‐sheet 34	A,E
G800W	D	5	20.8	β‐sheet 38	B
G800R	D	8	20.8	β‐sheet 38	B
L809P	D	4	31.7	β‐sheet 39	‐
G891R	E	5	40.7	Loop	B,C
L892P	E	11	41.8	Loop	B
R916C	E	16	29.8	β‐sheet 39	A
R916H	E	13	29.8	β‐sheet 39	A
L956R	E	8	24.8	β‐turn	A
F1000S	E	11	32.4	β‐sheet 47	‐
(b) Activity defective					
H72L	A	17	6.5	Active site	‐
D74E	A	11	5.0	Active site	‐
Y99H	A	9	18.5	α‐helix 3	‐
D102N	A	15	21.0	α‐helix 3	‐
G153V	A	19	14.9	Loop	‐
D159N	A	10	9.7	Loop	B,C
H200L	A	25	13.9	Loop	‐
H200N	A	53	13.9	Loop	‐
R229W	A	30	23.7	α‐helix 9	C,D
P263L	A	9	15.4	Loop	‐
S318L	A	32	9.3	Loop	‐
P379L	B	19	18.1	Loop	D
G420V	B	15	37.6	Loop	E
G451C	C	30	12.9	Loop	B,D
V457E	C	33	14.0	α‐helix 17	A
T745R	D	18	25.5	β‐sheet 33	‐
R950P	E	16	41.2	β‐sheet 47	‐
(c) Not classified					
L518P	C	N.D.	34.5	β‐sheet 15	D
G801D	D	N.D.	24.3	β‐sheet 38	B
R916S	E	N.D.	29.8	β‐sheet 45	A

Abbreviation: N.D., no data.

^a^ Relative activity (%) versus wild‐type counterpart (Stensland et al., 2015).

^b^ Distance of the mutated residue to the catalytic Zn2^2+^ atom.

^c^ ‐, no contact with another chain.

**FIGURE 1:**
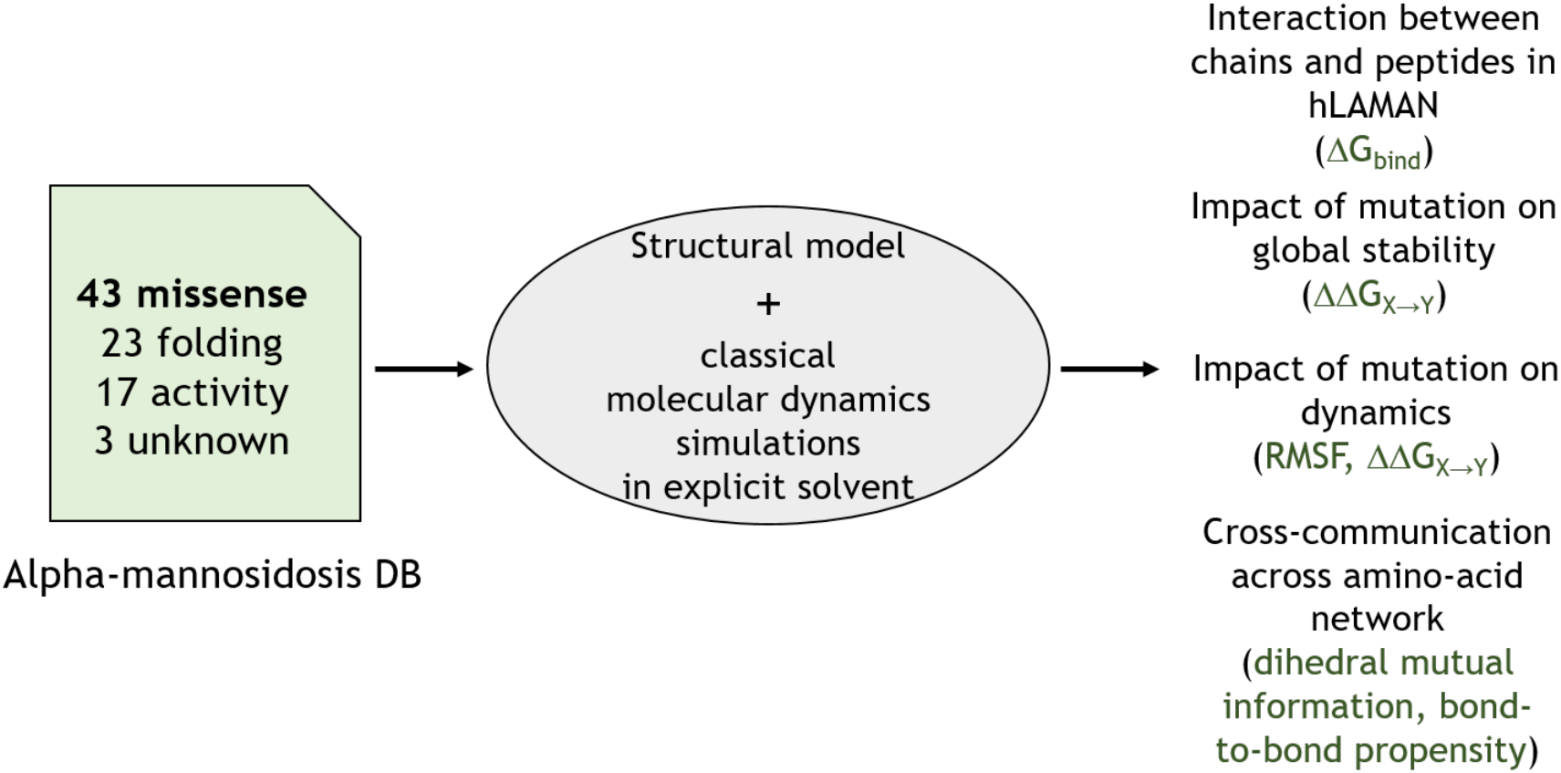
Analysis of hLAMAN MD ensemble to unveil the role of malignant missense mutations.

In this work, we have analyzed all‐atom conformational ensembles of hLAMAN generated via classical MD simulations in explicit aqueous solvent to unveil the key residues at the hLAMAN dimer interface and the molecular mechanism behind the reported missense mutations across the protein structure (Figure 1). In this way, we have been able to compute the degree of coupling of these mutations with the active site of the enzyme over its protein network, the impact of these mutations on the stability of the enzyme, and the role of enzyme dynamics. As an outcome, we have quantified each of the former factors for each of the reported mutations in hLAMAN. We show that by studying both the structure and the dynamics of enzymes in LSDs, new insights can be gained into the disease mechanism for future targeted therapies (Gil‐Martínez et al., 2022). Although the correlation between the type of missense mutation and the clinical manifestations is not evident in many LSDs, we believe our approach may help to shed light on this regard.

## 2 RESULTS AND DISCUSSION

### 2.1 Quaternary structure of hLAMAN

**FIGURE 2:**
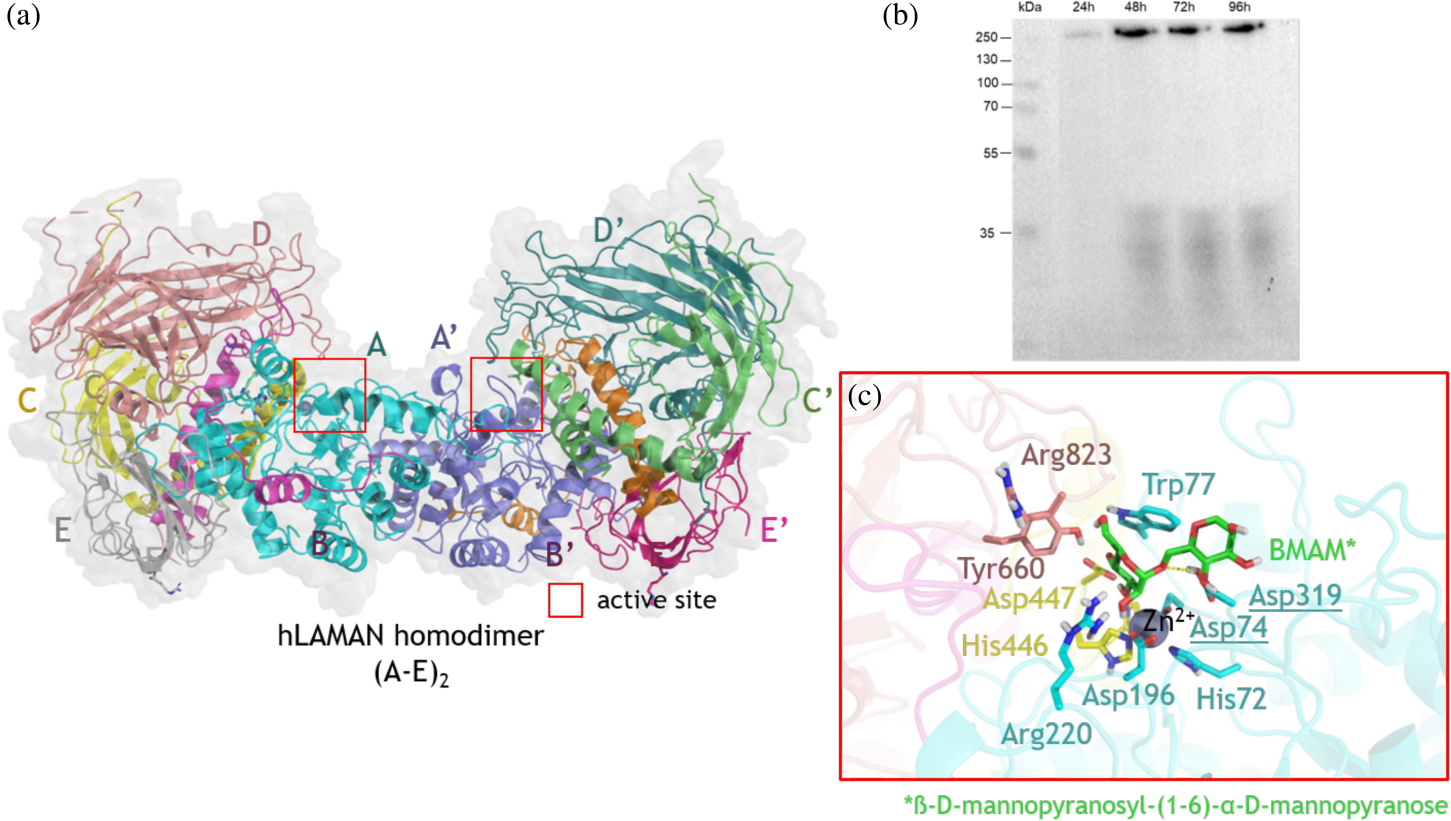
(a) hLAMAN quaternary structure. Peptides of the second polypeptide are labeled as A′–E′. (b) Western blot (gel run under native conditions) of hLAMAN expressed in *K. phaffii* for the indicated time periods. (c) Detail of the active site of hLAMAN in the presence of the substrate BMAM (β‐d‐mannopyranosyl‐(1,6)‐α‐d‐mannopyranose). Relevant residues at the active site are shown in sticks. The two catalytic acid residues are underlined.

We built up a model of the quaternary structure of hLAMAN as a polypeptide homodimer (A–E)_2_ based on experimental evidence (Figure 2a). The enzyme expressed in *Komagataella phaffii* appears as a single band at approx. 230 kDa in both PAGE gels (run under native conditions) and the respective Western blots. This corresponds to the predicted size of the hLAMAN homodimer of the polypeptides A‐E (Figure 2b). These results are in line with others (Liao et al., 1996) and previous size exclusion chromatography data (Berg et al., 2001). Noteworthy, we had difficulties observing this protein band in the secreted protein fraction (supernatant fraction). This fact may indicate the need for intracellular processing to complete the maturation of the protein. It is reported that each of the polypeptides of hLAMAN consists of five chains (named A‐E). hLAMAN is biosynthesized as a polypeptide of ca. 120 kDa in the endoplasmic reticulum (ER) and then transported to the lysosome (Hansen et al., 2004). Along this journey, the maturation of the hLAMAN polypeptide yields three fragments of approx. 70 (chains A, B, C cross‐linked via four disulfide bonds), 40 (chain D), and 15 (chain E) kDa, respectively. Additionally, the recombinant hLAMAN expressed in different eukaryotic heterologous systems (e.g., Chinese hamster ovary (CHO) cells (Berg et al., 2001), COS‐1 cells (Hansen et al., 2004), or *K. phaffii* (Liao et al., 1996)) may be secreted as the whole precursor polypeptide (120 kDa). Interestingly, this recombinant precursor evolves when concentrating the crude protein by cleavage between chains C and D, with the appearance of bands at ca. 70 and 55 kDa in SDS‐PAGE gels (Berg et al., 2001). Looking at the crystallographic symmetry in bLAMAN, the most plausible interaction between polypeptides in the homodimer of bLAMAN and hLAMAN ((A‐E)_2_) is via chains A and A' (Figure 2a). The hLAMAN structure consists of 23 α‐helices and 50 β‐sheets (Figure S1). The analysis of the molecular interfaces of our hLAMAN structure and the analogous structure of bLAMAN (PDB id. 1O7D) (Heikinheimo et al., 2003) with the PISA server (Krissinel & Henrick, 2007) points out that the most probable assembly would be the one corresponding to the quaternary structure of a single A‐E assembly (chains A‐E, Figure 2a). The active site of hLAMAN is located at the interface between chains A, C, and E within each A–E unit. It is composed of a Zn^2+^ atom coordinated by three residues of chain A (H72, D74, and D319) and one residue of chain C (H446). D74 and D319 on chain A act in catalysis as nucleophile and general acid, respectively (Figure 2c) (Suits et al., 2010). Interestingly, all these residues are located on loops between α‐helices (Figure S1).

**FIGURE 3:**
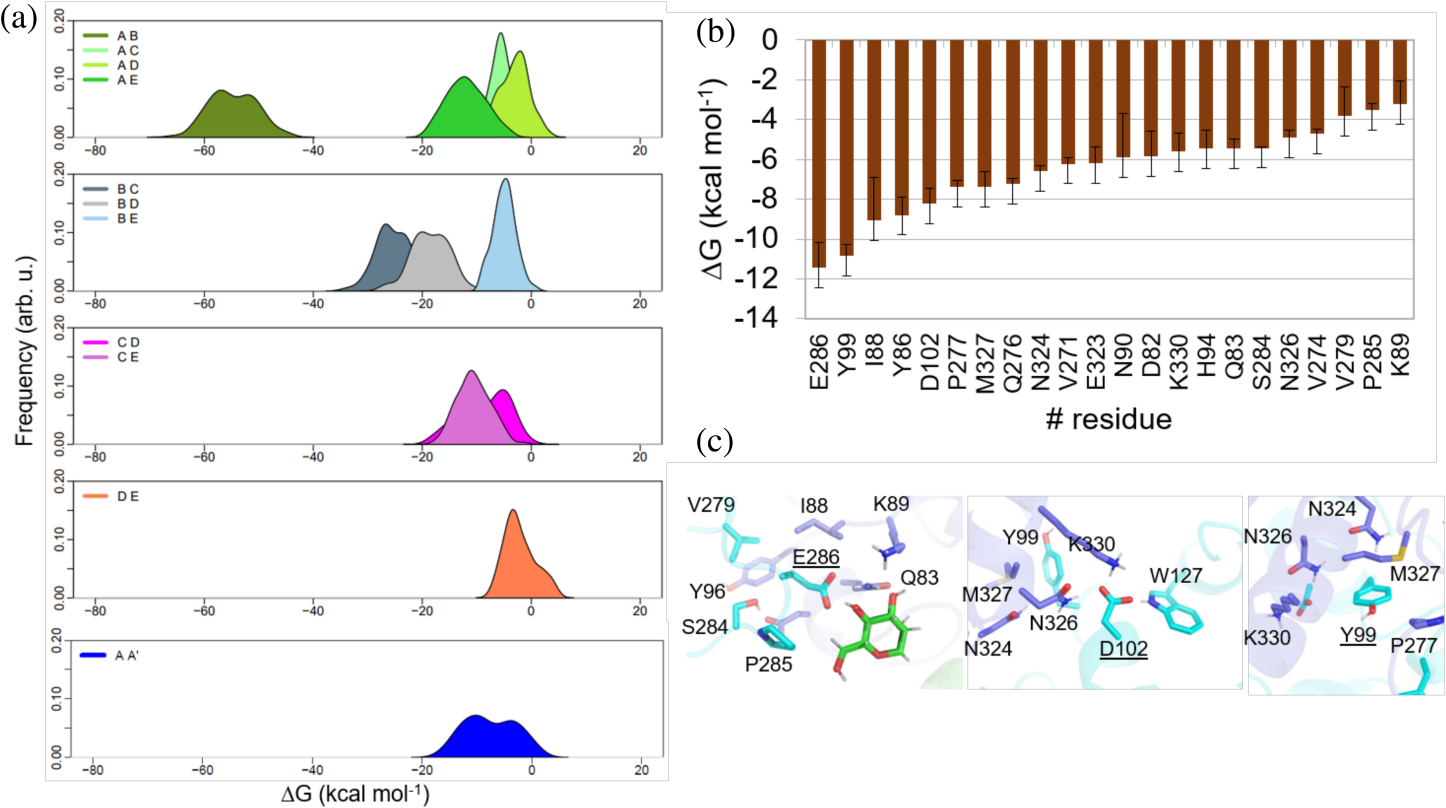
(a) Computed binding energy (kcal mol^−1^, ±SD) between peptide chains in hLAMAN along the MD simulations. (b) Relevant residues at the A–A′ interface and energy contribution per residue (kcal mol^−1^). (c) Details of the interactions of E286, D102, and Y99 at the homodimer interface of hLAMAN.

We studied the interactions between subunits in hLAMAN. To do this, we embedded hLAMAN into a box of water molecules and ran *μ*s‐scale molecular dynamics (MD) simulations of the system. We studied the energetic contributions between interfaces (see Methods for further details). In Figure 3a, the binding energy distributions between peptides A–E in the hLAMAN monomer computed along the MD simulation trajectories are shown. The interaction between peptides A and B has the largest energetic contribution to the stability of the protein complex, whereas the other cross‐interactions between chains (e.g., B–C, D–E, or C–D) contribute to a smaller extent. There are still some differences between them: whereas chain B has a strong interaction with chain C, the interaction between D and E, although favorable, has a much smaller contribution. In all these cases, their mean values for the interactions between A and C, B and E, or D and E are below 10 kcal mol^−1^.

We also explored the interactions of the polypeptide interface (A–A′ interface). The hLAMAN homodimer interacts mainly via the two A–A′ chains through helices H3 and H11. Indeed, a favorable interaction between A and A′ is observed along the MD trajectories (Figure 3b). Looking in detail at the individual energy contribution of the interfacial residues, it is found that E286, Y99, I88, Y86, D102, P277, M327, and Q276, among others, are the relevant ones for the stability of the homodimer (Figure 3b). The side chain of E286 establishes a salt bridge with K89 and a hydrogen bond with Q83. A hydrogen bond is also observed with an α‐d‐mannopyranose from the other subunit A'. Importantly, the carboxylic acid of the side chain of D102 (located on H3) establishes a hydrogen bond with the indole nitrogen of W127 (chain A), the amide nitrogen of the side chain of N326, and the ammonium group of K330. The latter two residues are located on the other chain A′ (Figure 3c). The Y99H enzyme variant has been detected in one patient (Riise Stensland et al., 2012), and D102N has also been listed within the missense mutations (Stensland et al., 2015). The mutation D102N causes a drop of the enzyme activity up to 85% of the wild‐type counterpart activity. The hydrogen‐bond‐acceptor character of both oxygen atoms of the D102 side chain is essential to keep these three interactions between A and A′. As a consequence, the introduction of an amide function would not make such an interaction network possible. The change Y99H also significantly reduces the enzymatic activity. The phenol ring in Y99 interacts with the side chain of M327. A hydrogen bond between the phenol oxygen of Y99 and the backbone of Y86 orients the phenol ring. Thus, the introduction of an imidazole in such a position may imbalance the polarity of this section of the interface. Altogether, E286, Y99, and D102 are in close proximity on helices H3 and H11. These regions are critical for the integrity of the hLAMAN homodimer.

### 2.2 Reported missense mutations and hLAMAN structure

So far, 43 deleterious missense mutations in hLAMAN have been reported in MANSA (Table 1) (Kuokkanen et al., 2011; Stensland et al., 2015). A first group of 23 mutations has been shown to prevent the protein folding of the enzyme in the ER or to have impaired transport to the lysosome; 17 of them reduce the activity of hLAMAN, but do not affect its folding, and 3 of them have not been classified yet. Looking at the location of the reported mutations affecting the activity on the structure of hLAMAN, it can be seen that most of them are at a distance of more than 10 Å away from the active site. Very few of the mutations (e.g., H72L, D74E, or P197R) are located in the proximity of the active site. This highlights the challenging nature of explaining the loss or reduction of enzyme activity by simple visual inspection of the protein structure.

### 2.3 Impact of the missense mutations on hLAMAN stability

**FIGURE 4:**
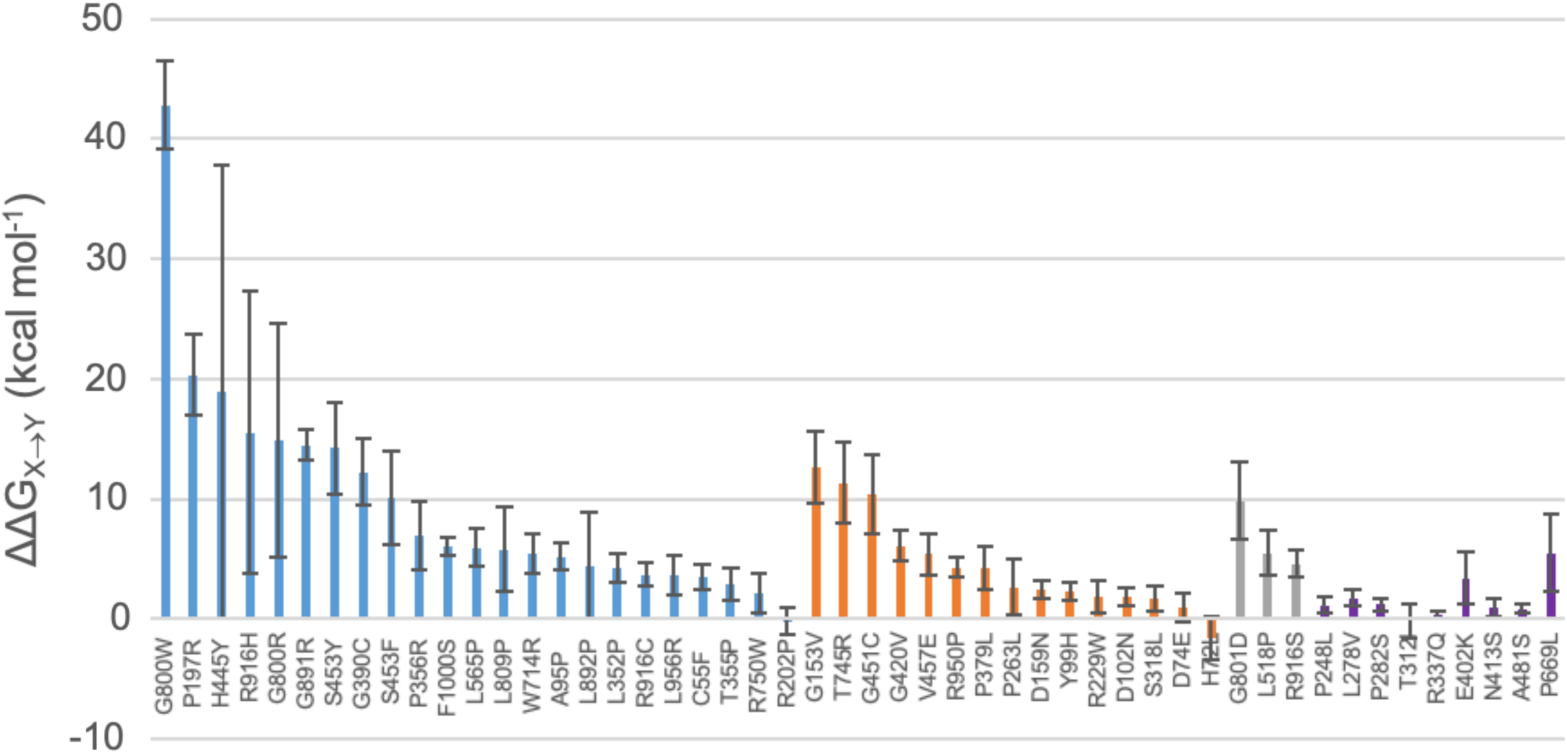
ΔΔ*G*_*X*→*Y*_ (kcal mol^−1^, ±SD) for the missense mutations affecting the enzyme folding (blue bars), the enzyme activity (orange bars), or of unknown effect (gray bars) computed using MD ensembles and FoldX5 (Schymkowitz et al., 2005). We also include nine benign mutations (violet bars) (Riise Stensland et al., 2012). Mutations affecting residue H200 present values above 50 kcal mol^−1^ and have been excluded.

Almost half of the reported missense mutations affecting the folding of the protein (13 out of 23) are located in ordered regions, while most of the mutations causing a decrease in enzyme activity (10 out of 17) are located in loops or disordered regions. This observation can be linked to the fact that changes in α‐helices or β‐sheets (ordered regions) can deeply alter the folding outcome in hLAMAN. To support this hypothesis, we measured the impact of each of the 23 missense mutations affecting the folding in hLAMAN on the global stability of the enzyme (Figure 4 and Table S1). We calculated the change of the free energy of the enzyme variant by replacing residue X by the deleterious mutation Y (as ΔΔ*G*_*X*→*Y*_, see Materials and Methods in Section 4). We computed ΔΔ*G*_*X*→*Y*_ using FoldX (Schymkowitz et al., 2005), which tend to detect the impact on the local environment of the mutation. By using conformational ensembles, we indirectly capture the impact of remote mutations on the network of interactions with active site residues, potentially accounting for conformational changes that affect enzyme activity upon mutation. In most of the folding mutations, the system increases its free energy significantly (median of 5.0 kcal mol^−1^, with 48% of them over ΔΔ*G*_*X*→*Y*_ > 10.0 kcal mol^−1^). This highlights the impact of these mutations on the secondary structure of hLAMAN. As an example, the changes on Pro and Gly residues has the larger impact on ΔΔ*G*_*X*→*Y*_ (e.g., G800 in chain D). In contrast to the former folding mutations, the values for the ones affecting the enzymatic activity, but not the folding exhibit smaller ΔΔ*G*_*X*→*Y*_'s (median of 6.00 kcal mol^−1^) and only a 39% of them are above 10 kcal mol^−1^. Only G153V, T745R, or G451C within this group decrease the stability of the protein scaffold. This may indicate that in the former group of mutations, the lack of the activity is not due to a loss of the structure of the protein, but rather to a change of the dynamics and the network of interactions within hLAMAN. Those mutations located at the active site (H72L or D74E) have a residual impact on the global stability of the protein scaffolds, with ΔΔ*G*_*X*→*Y*_ close to zero or even negative. In addition to that, the mutations Y99H and D102N, affecting two positions important for the interface between the chains A and A′ in the oligomer (Figure 3), show very low ΔΔ*G*_*X*→*Y*_. These data agree well with the experimental evidence that Y99H is transported to the lysosome (Kuokkanen et al., 2011), suggesting sufficient folding in the ER. Regarding the mutations that have not been classified, only G801D shows a value close to 10 kcal mol^−1^.

We also computed the ΔΔ*G*_*X*→*Y*_ values for 9 benign mutations detected in patients (violet bars in Figure 4) (Riise Stensland et al., 2012): P248L, L278V, P282S, T312I, R337Q, E402K, N413S, A481S, and P669L. These enzyme variants show 37%–124% of wild‐type activity when using COS‐7 or BHK‐21 cells. Most of the benign mutations have a value of ΔΔ*G*_*X*→*Y*_ close to zero, with E402K and P669L far from the value of 10 kcal mol^−1^. Finally, we ran a Mann–Whitney *U* test analysis to check if we can differentiate between the two groups of mutations (folding, activity) based on their the ΔΔ*G*_*X*→*Y*_ values (Table S2). No statistical significant difference between folding and activity mutation groups is found at a significant level of 5% (*p*‐value = 0.080).

### 2.4 Impact of the missense mutations on the dynamics of hLAMAN

**FIGURE 5:**
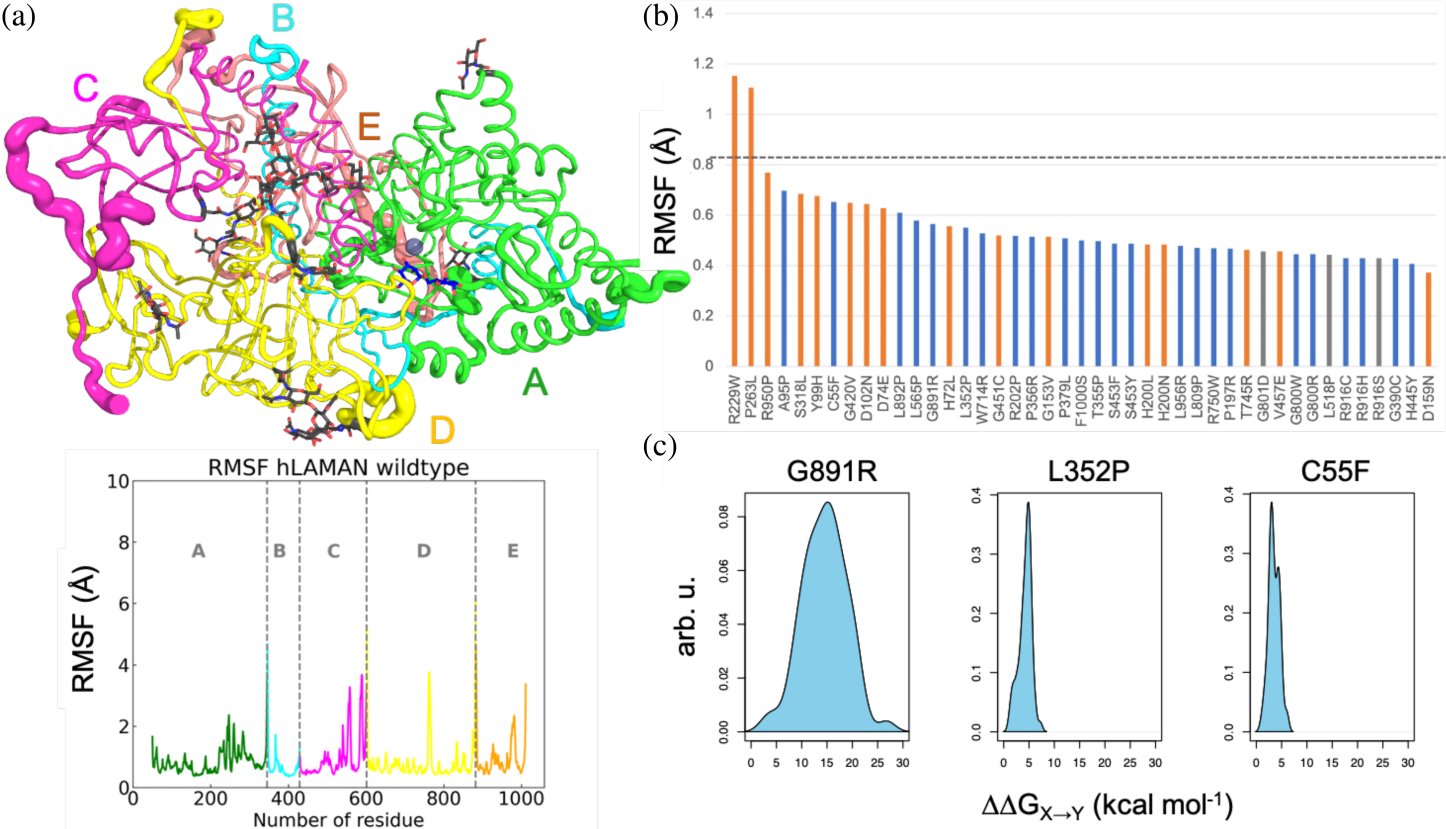
(a) Root‐mean‐square fluctuation (RMSF, Å) in MD simulations and dynamical regions on wild‐type hLAMAN. Peptides A–E are shown. (b) RMSF values for the reported missense mutation positions. The gray‐dotted line highlights the RMSF mean value in hLAMAN. (c) ΔΔ*G*_*X*→*Y*_ (kcal mol^−1^) distribution of G891R, L352P, and C55F in MD simulation.

We further analyzed the dynamics of hLAMAN in MD simulations. The core globular domain of hLAMAN shows low dynamical fluctuations along the trajectories, as can be seen in the root‐mean square fluctuation (RMSF) values per residue (mean RMSF of 0.83 Å, Figure 5a). In contrast, the β/γ‐loops exposed to the solvent (e.g., residues 539–543, 551–562, 583–593, 759–768, and 983–985) and the spliced regions between peptide chains (A,B: K345‐G346; C,D, S600‐W601; D,E, G882‐A883) have a noticeable mobility. The computed RMSF values on MD trajectories show that positions R229 and P263 move significantly along the MD trajectory (Figure 5b and Table S3). Whereas P263 is located in a loop, R229 is placed in the α‐helix H9. However, since we analyze MD snapshots derived from the wild‐type hLAMAN scaffold, the impact of the reported mutations on the dynamics of the new enzyme variants is difficult to anticipate by means of the wild‐type RMSF values. In addition to that, most of the *loci* for the described mutations have an RMSF value below the mean value (Figure 5b). Therefore, we decided to look for alternative descriptors. Since we are using conformational ensembles of hLAMAN, it is possible to quantify the impact of the residue substitution on the dynamics of the protein. The differential width of the former ΔΔ*G*_*X*→*Y*_ energy distribution for each of the explored mutations is related to the dynamics of the local environment (Table S1). As an example, the change G891R on the β42–β43 loop of subunit E shows a broad ΔΔ*G*_*X*→*Y*_ (standard deviation of 4.38 kcal mol^−1^), whereas the change of L352P in the β‐sheet 9 of subunit B has a much smaller bandwidth (standard deviation of 1.24 kcal mol^−1^, Figure 5b). Indeed, for some residues (e.g., C55F removing the disulfide on chain A), more than one maximum in ΔΔ*G*_*X*→*Y*_ is found, which illustrates different conformational states of hLAMAN in MD simulations. Compared to the RMSF values, the standard deviation in ΔΔ*G*_*X*→*Y*_ can be used as a measure of the impact of the missense mutation in the dynamics of the local environment using MD ensembles of the wild‐type enzyme.

### 2.5 Coupling of the remote missense mutations with the active site

**FIGURE 6:**
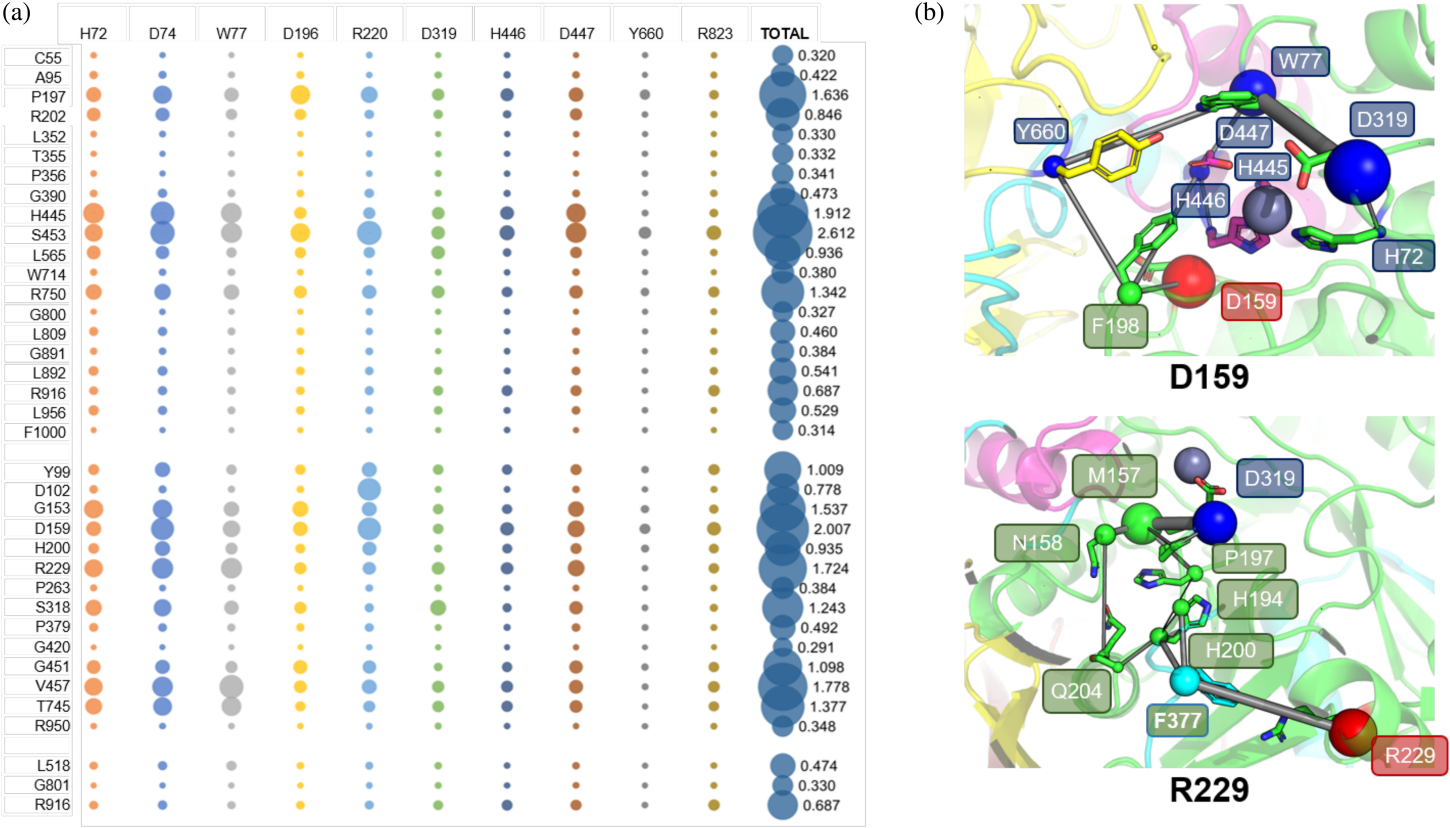
(a) MI values of the reported missense mutations with 10 residues at the active site. No data are shown for H72 and D74 as they are included into the 10 residues of the active site. The last column refers to the total MI value for each mutation with the 10 residues at the active site. The larger the bubble, the larger the coupling. (b) Paths between reported mutations (source, shown as red spheres) and D159 and R229 (sink, shown as blue spheres) calculated via WISP. The connecting nodes are also as spheres of different size.

As we commented before, most of the reported mutations are not in the proximity of the active site of the enzyme. But how do they affect the enzyme activity if they are located remotely? In order to answer this question, we interrogated our MD trajectory data to quantify the degree of “cross‐talk” between residues in the protein. We computed the mutual information (MI) between each of the reported positions containing a mutation and 10 residues at the active site (Fleck et al., 2016) (Figure 6 and Table S4). Those mutations that are located at positions at the active site (H72 and D74) are not included in the analyses. MI refers to the dependency between two variables or elements, like residues in this case. We use as metrics the total pairwise MI between all dihedral angles (including 𝜙 and *ψ*) of 10 residues from the active site and the dihedral angles from the rest of the residues of the protein scaffold. The larger the MI, the stronger the communication between residues. In general, we observe a strong coupling of the reported positions with three residues at the active site: D74, R220, and to a lesser extent, W77. In contrast, there is a weak coupling with Y660. Within the group of mutations affecting the activity of the enzyme, 64% of them have a total dihedral MI value above the mean value from all residues (0.867). D159 is the one with the highest total dihedral MI value with the residues of the active site (mainly via D74 and R220). V457 and R229 are the following residues with the larger dihedral MI values within this group of mutations. In the case of mutations affecting the folding of the enzyme, only 25% of the mutations show a total dihedral MI value above the mean MI value, with positions S453 and H445 as the ones with larger coupling to the active site. Very weak coupling is observed for residues P379, P263, R950, and G420 in the group of defective enzymes and P356, L352, T355, G800, C55, and F1000 in the missense mutations affecting the folding. We also ran a Mann–Whitney *U* test analysis to check if we can differentiate between the MI values of the two groups of mutations (Table S5). Although we see that the group of mutations affecting activity tend to have larger MI values, no statistically significant difference between folding and activity mutation groups is found at a significant level of 5% (*p*‐value = 0.139).

Furthermore, we studied the pathways between the missense mutations and the residues at the active site using the weighted implementation of suboptimal path (WISP) analysis (Crean et al., 2023) (see Material and Methods). Since the folding mutations may prevent the quaternary structure of hLAMAN, we focused our analyses on the positions of the reported mutations affecting enzyme activity. In Figure 6b, the WIPS trajectories for two of the strongly coupled residues with the active site are represented: D159 and R229. The side of the spheres denotes the frequency of a node (residue), and the width of the cylinders denotes the frequency of an edge (residue–residue interaction) in the generated path. Larger sizes indicate higher frequencies, which means an increased importance in allosteric communication. The strongly coupled position D159 only needs four residues to reach the active site (77, 159, 198, and 445). In contrast, the residue R229 needs at least eight residues to reach it. On the way to the active site, other positions listed in Table 1 (e.g., H200) are found. The pathways for the rest of the positions of the missense mutations affecting the activity are plotted in Figures S3 and S4, summarized in Table S6, and the contributions of the node in Figure S5.

**FIGURE 7:**
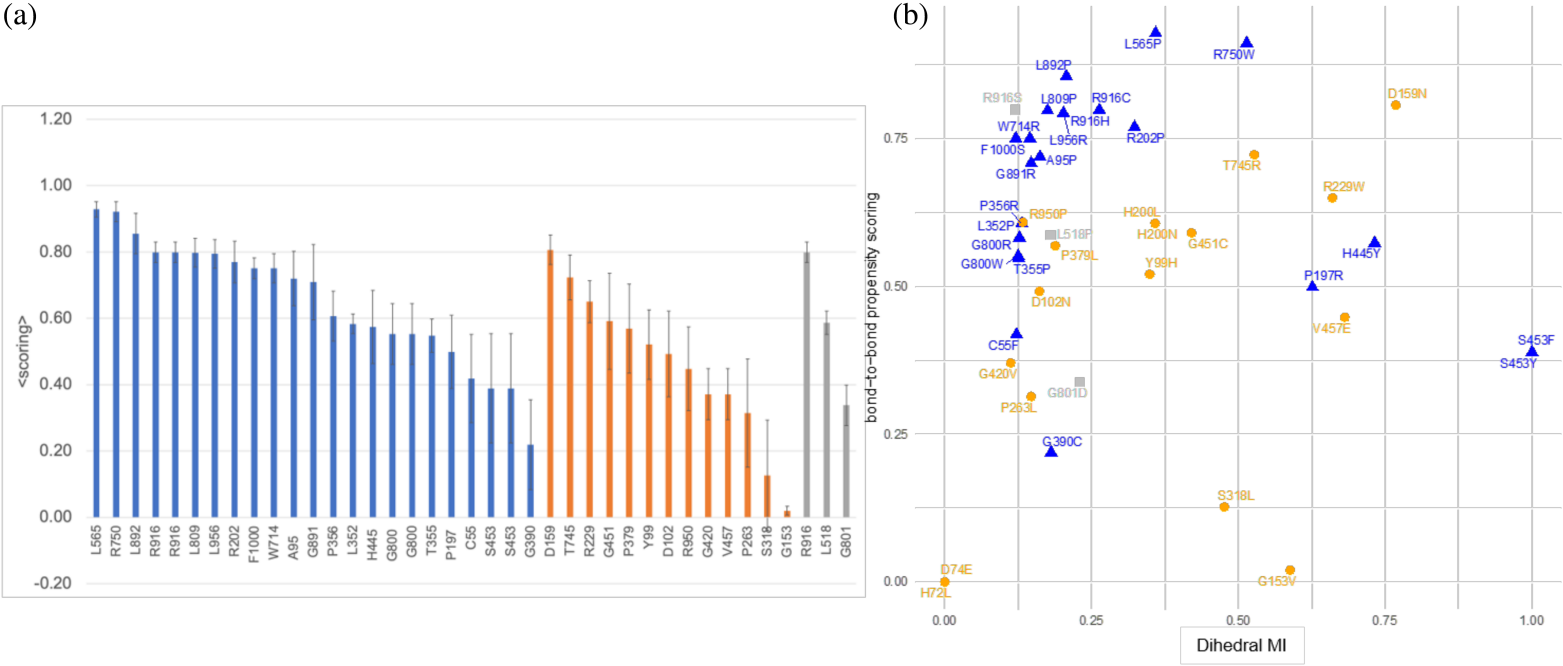
(a) Scoring for bond‐to‐bond propensities for the positions. (b) Total dihedral MI versus bond‐to‐bond propensity scoring for the reported missense mutations.

We further analyzed the coupling of the reported mutations with the same 10 residues from the active site but via bond‐to‐bond propensity descriptor (Figure 7a and Table S2) (Amor et al., 2014; Hodges et al., 2018). Bond‐to‐bond propensity describes the effects of fluctuations of given bonds on any other bond in the protein. As before, the bond‐to‐bond scoring is defined between 0.0 and 1.0, but in this case, 1.0 is the maximum value for each position. In contrast to the dihedral‐dihedral MI analysis, with the bond‐to‐bond propensity, we analyze the interaction between residue via an energy‐weighted graph representation of the protein structure. Positions related to the misfolding of hLAMAN have in general high bond‐to‐bond propensity scoring values. The ones related to the loss of the activity of the enzyme tend to have smaller values. More of the half of the positions of the misfolding group shows a mean scoring value above 0.60. In contrast, only 4 positions out of 12 have a mean value ≥0.60 in the group of mutations affecting the enzyme activity. For the non‐characterized missense mutations, the positions R916 and L518 show large scoring values (≥0.60). G801 presents a small value. We represented the total dihedral MI versus the bond‐to‐bond scoring mean values (Figure 7b). We normalized all the data by dividing by the maximum value within the series. This way, all data are distributed within {0,1}. Few positions (D159, R229, R750, and P197) present both high MI and bond‐to‐bond scoring values (right top quadrant). In the left top quadrant (low total dihedral MI value and large bond‐to‐bond scoring), most of the positions related to folding missense mutations are located, with only two mutations affecting the activity (P379 and G451), which are glycine/proline residues. Regarding the position of the missense mutations that are not classified yet, two of them (R916 and L518) are present in this quadrant, while G801 presents both low MI and bond‐to‐bond scoring. Altogether, with this projection of MI versus bond‐to‐bond scoring, a tendency is observed for the missense mutations affecting the folding of the enzyme: low MI values and large bond‐to‐bond propensities.

### 2.6 A new view on hLAMAN missense mutations

**FIGURE 8:**
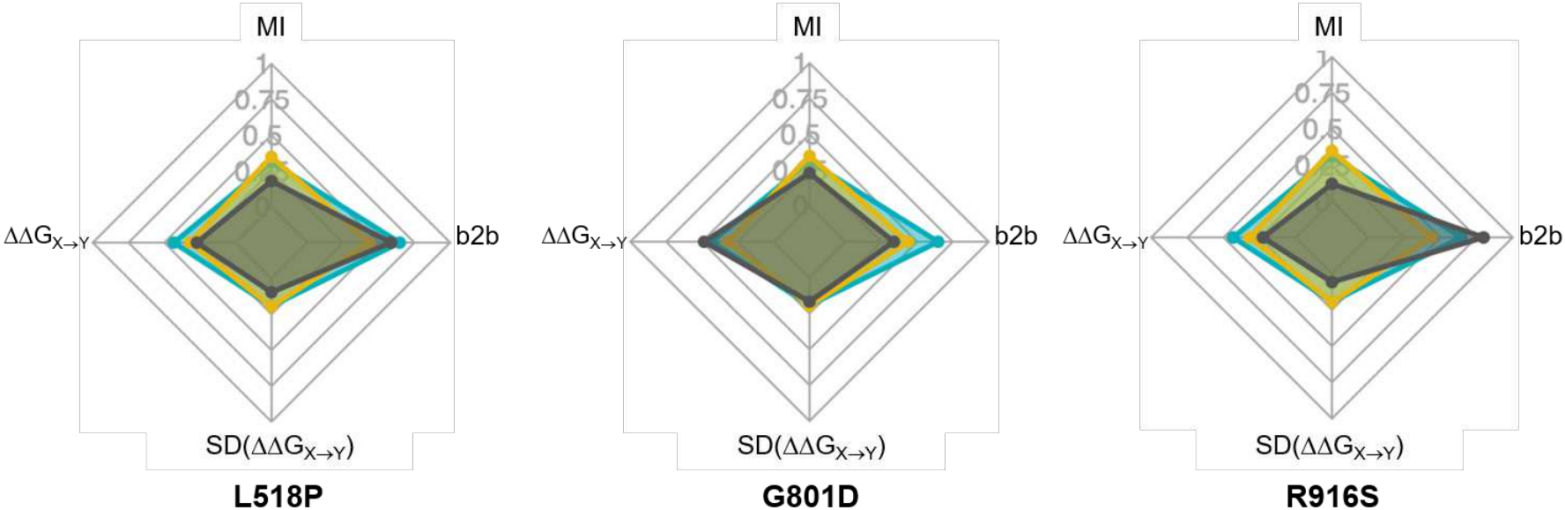
Radar chart of variants L518P, G801D, and R916D (gray surface) for the descriptors total dihedral MI, bond‐to‐bond scoring (b2b), ΔΔ*G*_*X*→*Y*_, and the standard distribution of the latter. All values are normalized and defined between 0 and 1. The median values of the defective activity (orange surface) and folding (blue surface) missense groups are also shown.

The analysis of MD‐derived conformational ensembles can provide useful information about the molecular basis for each of the reported mutations. As discussed above, we have analyzed the impact of the reported mutation by computing from conformational ensembles the stability of the enzyme (ΔΔ*G*_*X*→*Y*_ values), the cross‐talking of each of the positions with the active site (total dihedral MI and bond‐to‐bond propensity scoring), as well as the dynamical component for each of the positions (width of the ΔΔ*G*_*X*→*Y*_). The different scoring of each of the residues in each of these four descriptors can unveil the molecular basis for causing malignancy. We merged the data of the four descriptors for all missense mutations and we plotted the data as a radar chat (Table 2 and Figure S6). All values are defined for a range between 0 and 1 after normalizing by the maximum value of the two series. Looking at Table 2, the relative impact of one mutation in each of the descriptors can be identified. In the graphical representation, the radar chart of the mutations can provide a quick overview of the molecular reasons for malignancy for a given variant. As an example, in Figure 8 the three unknown variants, which have not yet been classified, are shown: L518P, G801D, and R916D. The median values for the folding and defective activity variants are also shown. Based on their projections, the G801D enzyme is more similar to those mutations causing unfolding, whereas L518P and R916S mimic the radar chart of the group of mutations causing the loss of enzyme activity, with a strong coupling with the active site via the backbone bones. Of the three mutations, G801D is the one with the larger impact on the stability of hLAMAN and L518D the one with a larger dihedral coupling with the active site (total dihedral MI values).

**TABLE 2:** A new analysis of the reported missense mutations in hLAMAN analyzing MD ensembles.

Mutant	Chain	Total dihedral MI	ΔΔ*G*_*X*→*Y*_	SD (ΔΔ*G*_*X*→*Y*_)	Bond‐to‐bond propensity
(a) Protein folding					
C55F	A	0.122	0.172	0.056	0.419
A95P	A	0.162	0.257	0.058	0.720
P197R	A	0.626	1.000^a^	0.178	0.499
R202P	A	0.323	0.011	0.059	0.770
L352P	A	0.127	0.209	0.066	0.583
T355P	B	0.126	0.141	0.071	0.548
P356R	B	0.131	0.343	0.152	0.607
G390C	B	0.181	0.603	0.145	0.219
H445Y	C	0.732	0.929	1.000	0.574
S453Y	C	1.000	0.495	0.205	0.389
S453F	C	1.000	0.495	0.205	0.389
L565P	C	0.359	0.294	0.087	0.929
W714R	D	0.145	0.270	0.087	0.751
R750W	D	0.514	0.106	0.085	0.912
G800W	D	0.125	1.000^a^	0.192	0.552
G800R	D	0.125	1.000^a^	0.515	0.552
L809P	D	0.175	0.285	0.186	0.798
G891R	E	0.147	0.714	0.066	0.709
L892P	E	0.207	0.220	0.232	0.856
R916C	E	0.263	0.182	0.052	0.799
R916H	E	0.263	0.764	0.620	0.799
L956R	E	0.202	0.182	0.085	0.794
F1000S	E	0.121	0.295	0.040	0.751
(b) Activity defective					
H72L	A	0.000^b^	0.077	0.094	0.000^b^
D74E	A	0.000^b^	0.048	0.065	0.000^b^
Y99H	A	0.349	0.116	0.039	0.521
D102N	A	0.161	0.091	0.038	0.492
G153V	A	0.588	0.623	0.157	0.020
D159N	A	0.768	0.122	0.043	0.807
H200L	A	0.358	1.000^a^	1.000^a^	0.607
H200N	A	0.358	1.000^a^	1.000^a^	0.607
R229W	A	0.660	0.092	0.072	0.650
P263L	A	0.147	0.132	0.120	0.314
S318L	A	0.476	0.085	0.057	0.127
P379L	B	0.188	0.208	0.096	0.569
G420V	B	0.112	0.301	0.069	0.371
G451C	C	0.420	0.514	0.175	0.591
V457E	C	0.681	0.266	0.091	0.448
T745R	D	0.527	0.559	0.179	0.723
R950P	E	0.133	0.213	0.043	0.609
(c) Not classified					
L518P	C	0.180	0.272	0.096	0.587
G801D	D	0.230	0.485	0.168	0.338
R916S	E	0.120	0.227	0.058	0.799

^a^ Data above 20.35 kcal mol^−1^ is the normalizing value.

^b^ Residue defined as part of the active site.

Finally, it is important to emphasize that while we have established a metric to provide molecular insights into the reported deleterious mutations, the use of conformational ensembles of the wild‐type enzyme to predict the impact of point mutations on enzyme structure still has inherent limitations. When relying on a fixed configuration, it becomes challenging to detect allosteric rearrangements or fully capture the structural response upon remote mutations. Moreover, employing a force field like FoldX to compute ΔΔ*G*_*X*→*Y*_ values does not account for long‐range interactions.

## 3 CONCLUSIONS

We have analyzed MD‐derived conformational ensembles to understand how remote missense mutations affect the loss of enzyme activity and the folding of hLAMAN. The relevant biological quaternary structure of hLAMAN is a homodimer of the polypeptides A‐E. By analyzing the key residues for the integrity of the dimer interface between units A and A' in hLAMAN, we could assign an important role to the reported missense mutations like Y99H and D102N. They are located at the hLAMAN homodimer interface, and they participate in a well‐defined amino acid network. Thus, their mutation may significantly disturb this network of interactions between both units. Additionally, the interaction between units A and B in the polypeptides A–E of hLAMAN is the stronger one among all subunits. The computation of the impact of the missense mutation on the global stability of the enzyme scaffold as ΔΔ*G*_*X*→*Y*_ shows that the mutations affecting the folding of hLAMAN present larger values than the group affecting the activity, although we cannot differentiate statistically between the two groups. On the contrary, the analysis of 9 benign/neutral mutations shows how their ΔΔ*G*_*X*→*Y*_ are close to zero. In addition to that, the fluctuations of ΔΔ*G*_*X*→*Y*_ along the MD ensembles indicate the relevance of the mutation on the interactions within the local environment and its dynamical effect. The use of RMSF per residue along the MD simulation does not provide such information. We have also analyzed the coupling of the reported mutations with 10 residues located at the active site via two metrics: the total dihedral MI and the bond‐to‐bond propensity scoring. Using them, we see that the two groups of missense mutations show different trends for each of the descriptors, what could be used as a classification metrics. Altogether, we have reported here the contribution of each of the pathogenic missense mutations into the stability of hLAMAN, its dynamics, and the network of interactions with the active site. Such analyses and understanding at the atomic level can help in deciphering the role of future missense mutations in LSDs as well as in better adapting and designing novel molecules (i.e., pharmacological chaperones or ERTs), and potentially in contributing to the foundation of precision medicine in LSDs.

## 4 MATERIALS AND METHODS

### 4.1 hLAMAN expression and purification

The gene encoding hLAMAN (MAN2B1) was acquired and inserted into the pET28b + vector (GenScript). After amplification using the primers 5′‐TGCGCGCACGTGGATGGGCAGCAGCCATCATCATCATCATCACAG‐3′ and 3′‐TGCGCGGCGGCCGCTTAGCCATCCACTTCC‐5′ (GenScript), the gene of interest was cloned into the *K. phaffii* expression vector pPICZalpha (Thermo Fischer Scientific) using the restriction enzymes EcoRI and NotI. Following transformation (100 ng of DNA plasmid) and amplification in *E. coli* NEB10‐beta cells, the final pPICZalpha was isolated and 13 μg of the plasmid DNA was linearized using SacI as the restriction enzyme. *K. phaffii* X33 was transformed with the linearized plasmid. Positive colonies were selected on YPDS agar plates containing 100 *μ*g mL^−1^ of the antibiotic Zeocin*™*. To test the efficacy of the hLAMAN expression, a high‐throughput expression screening was performed by cultivating different colonies in 96‐deep well plates followed by dot blotting. The 96‐deep well plates containing a minimal glucose medium in 0.4 M potassium phosphate buffer pH 6.0 (1.34% yeast nitrogen base, 1% d‐glucose, 500× biotin) were inoculated with 80 different colonies and grown in the presence of different concentrations of Zeocin*™* (100, 250, 500, and 1000 μg mL^−1^). hLAMAN expression was induced by the addition of methanol (5% methanol in glucose‐deprived minimal medium) every 24 h. After 96 h, cells were harvested by centrifugation, and the protein fractions from the supernatant were subjected to dot blotting on a nitrocellulose membrane. After blocking the membrane in 5% (w/v) non‐fat dry milk in 1x Tris‐buffered saline, 0.1% Tween® 20 Detergent (TBST) buffer at pH 7.4, anti‐HIS IgG (diluted in 5% (w/v) non‐fat dry milk in TBST) was used as the primary antibody. After 2 h of incubation, the membrane was washed four times with 1× TBST and then incubated with HRP‐conjugated goat‐anti‐rabbit IgG (diluted in TBST containing non‐fat dry milk (5%, (w/v))) for 1 h. After another four washing steps with 1× TBST, the immunoreactive bands were visualized by chemiluminescence detection using the Super Signal West Femto Maximum Sensitivity substrate (Thermo Fischer Scientific) and a Chemidoc detection system (BioRad). The colony showing the highest expression level was selected for a scale‐up expression. 25 mL of BMGY media (1% yeast extract, 2% peptone, 100 mM potassium phosphate buffer, pH 6.0, 1.34% YNB, 500x biotin, 1% glycerol) was inoculated with a single colony and incubated overnight at 28°C under gentle shaking. At an optical density at 600 nm of 6.0, the cells were harvested and resuspended in 200 mL of BMMY media (1% yeast extract, 2% peptone, 100 mM potassium phosphate buffer, pH 6.0, 1.34% YNB, 500× biotin, 0.5% methanol) to induce enzyme expression. To maintain expression, 100% methanol was added every 24 h to a final concentration of 0.5% at 28°C. Aliquots were collected at various time points, centrifuged, snap‐frozen, and stored for expression analysis of the pellet and the supernatant using SDS‐PAGE and Western blot using anti‐His IgG (Abcam, ab9108).

### 4.2 Modeling of the quaternary structure of hLAMAN

The initial Cartesian coordinates of the polypeptide of hLAMAN (UniProtKB ‐ O00754) were taken from the AlphaFold Protein Structure Database (AF‐O00754‐F1) (Jumper et al., 2021). The signal peptide (1–49 amino acids) was removed, and the loops were manually cleaved between amino acids 345–346 (A,B), 429–430 (B,C), 601–602 (C,D), and 882–883 (D,E) (Figure S1). The reported N‐glycosylation points at positions Asn137, Asn367, Asn497, Asn645, Asn692, Asn766, and Asn930 as well as the catalytic zinc atom, were placed into hLAMAN by structural superimposition with bLAMAN (PDB id. 1O7D^30^, UniProt code Q29451) or by manual docking. The experimentally reported disulfide bonds for the pairs 55–358, 268–273, 412–472, and 493–5019 were already present in the AlphaFold model and were included when building up the topology of the system. The protonation states of the titratable residues on the protein were calculated via the H++ web server (Gordon et al., 2005) assuming a pH value of 4.8 (close to the lysosomal one). We were careful with the catalytic residue Asp319 (general acid for the first step of the reaction), which was manually protonated to accomplish the proposed mechanism of action (Petersen et al., 2010). As hLAMAN substrate, we included the β‐d‐mannopyranose‐(1–6)‐α‐d‐mannopyranose (BMAM), derived from the crystal structure of a GH125 1,6‐α‐mannosidase variant (PDB id. 5M7I (Alonso‐Gil et al., 2017)). BMAM was manually docked into the active site of hLAMAN guided by the structural superimposition of GH125.

### 4.3 Classical molecular dynamics (MD) simulations

MD simulations were carried out using the suite of programs Amber20 (Case et al., 2022). Protein residues and solvated ions were treated with the AMBER ff19SB force field and GLYCAM06 for the N‐glycosylated residues and the substrate BMAM (Kirschner et al., 2008; Tian et al., 2020). Parameters for all atoms involved in the coordination sphere of the catalytic Zn^2+^ were derived by means of the Metal Center Parameter Builder (MCPB) approach (Li & Merz, 2016). The necessary quantum geometry optimization of the Zn^2+^‐coordination sphere in the gas phase was carried out with Gaussian16 (Frisch, 2016) at the B3LYP/6‐31G* level of theory (Becke, 1993; Lee et al., 1988; Stephens et al., 1994; Vosko et al., 1980). The hLAMAN monomer (A–E) as well as the hLAMAN dimer (A–E)_2_ were simulated. All systems were first energetically minimized to avoid steric clashes, and then placed in the center of a cubic box filled with 28,000–29,000 TIP3P water molecules (Mark & Nilsson, 2001). Then, the solvated systems were minimized in three consecutive steps (first all protons, then solvent, and finally, all system) and heated up in 50 ps from 100 to 300 K using an NVT ensemble with the Langevin thermostat (gamma friction coefficient of 1.0 ps ^−1^). Care was taken to constrain the solute during the heating step by imposition of a harmonic force on each atom of the solute of 40 kcal mol^−1^ Å^−2^. Afterward, these harmonic constraints were gradually reduced up to a value of 10 kcal mol^−1^ Å^−2^ in 4 simulation stages (NVT, 300 K). The systems were switched to constant pressure (NPT scheme, Berendsen barostat 300 K), and the imposed constraints from the heating were totally removed. Each of the systems was further simulated for a total time of 1.5 *μ*s in three independent MD simulations of 0.5 *μ*s. Atom‐pair distance cutoffs were applied at 10.0 Å to compute the van der Waals interactions and long‐range electrostatics by means of the Particle‐Mesh Ewald (PME) method. The SHAKE algorithm was applied to restrain the hydrogen atoms on water molecules (Miyamoto & Kollman, 1992). MD trajectory analysis was carried out using the *cpptraj* (Roe & Cheatham, 2013) module from Amber Tools23 (Case et al., 2023) for monitoring the root‐mean‐square deviation (RMSD) and root‐mean‐square fluctuation (RMSF), among other parameters.

### 4.4 Mutual information (MI) calculation

We considered the communication of all residues in hLAMAN with the active site via the torsional angles. We computed the mutual information (MI) pairwise values using the Parallel Software Suite for the Calculation of Configurational Entropy in Biomolecular Systems (PARENT) (Fleck et al., 2016; Fleck et al., 2018; Fleck & Zagrovic, 2019). As an input data set, we analyzed a merged MD ensemble from three independent MD simulations of hLAMAN monomer in explicit aqueous solution (3 × 0.5 *μ*s, 12,000 snapshots; the first 10 ns of the MD trajectories were not taken into consideration). All glycosylated residues were changed to Asn for the analysis afterwards. We analyzed the dihedral–dihedral MI values, which can present values from 0 (no coupling) to 1 (maximum coupling). These dihedral angles span all dihedrals, including the ones of the backbone (𝜙, *ψ*). In particular, we analyzed the torsional MI values between each residue *i* in the protein with 10 selected residues placed at the active site, as done in the bond‐to‐bond propensity analysis (H72, D74, W77, D196, R220, D319, H446, D447, Y660, and R823). A 962 × 962‐contact matrix was generated with the MI values (see Figure S2). The individual mean MI value across the matrix is 0.05, with auto‐coupling between amino acids excluded from the analysis. The mean MI value per residue with the 10 selected residues of the active site is 0.867.

### 4.5 Bond‐to‐bond propensity analysis

The bond‐to‐bond propensities for wild‐type hLAMAN were computed using the server ProteinLens (Amor et al., 2016; Mersmann et al., 2021b). The connectivity of the covalent bonds between the source (in our case the active site) and every other bond in the protein structure was measured with the bond‐to‐bond propensity analysis. This is done by assessing the propagation of a perturbation originating at the source site through an edge formulation of random walks. On their way, the propagation of these “fluctuations” within the model depends on the strength of interactions between atoms (e.g., hydrogen bonds, hydrophobic interactions). We utilized 9 randomly selected snapshots from the MD simulation of the wild‐type hLAMAN, excluding the first 100 ns of each of the single trajectories. As the primary source site for fluctuation propagation, we defined 10 residues located at or in close proximity to the active site: H72, D74, W77, D196, R220, D319, H446, D447, Y660, and R823. As a result, the statistical quantile score is obtained, which ranges between 0 (no coupling) and 1 (full coupling).

### 4.6 Weighted implementation of suboptimal path (WISP) analysis

WISP transforms the protein into a network and identifies how distal regions communicate allosterically. By designating a source and sink residue, the analysis reveals the shortest pathways for communication between them, pinpointing key residues and residue interactions. A correlation cutoff of 0.25 (absolute value) is used to determine the 500 shortest paths between source and sink, enabling the analysis of node and edge degeneracies alongside path lengths. We used the Key Interaction Finder (KIF) software (Crean et al., 2023). KIF methodology is based on the calculation of a correlation matrix between each of the structural features in the protein, which is transformed into a per‐residue matrix by identifying the largest correlation between any pair of residues. This step increases the dimensions from the number of features squared to the number of residues squared, with the diagonal set to 1. Next, the contact maps/matrices are created using the MDTraj library (McGibbon et al., 2015) to measure the heavy‐atom distances between residue pairs in a given structure. A 6 Å cutoff defines whether two residues are in contact, resulting in a binary matrix containing 1 (for contacts) and 0 (no contacts within the cutoff). These correlation and contact matrices are input into the R package Bio3D (Grant et al., 2006), facilitating the WISP analysis.

### 4.7 Binding energy and ΔΔ*G*_*X*→*Y*_ calculations

A total of 60 frames from the merged MD ensemble were printed and analyzed with the force field FoldX5 v2024 (Schymkowitz et al., 2005) to compute the binding energy between hLAMAN subunits (*AnalyseComplex* module) and to calculate the impact on the energy of the system after amino‐acid mutation as ΔΔ*G*_*X*→*Y*_ (*PositionScan* module). To calculate the individual energetic contribution of each of the resides at the A–A′ interface, we made use of the software MM‐ISMSA (Klett et al., 2012).

## AUTHOR CONTRIBUTIONS

**Špela Mandl:** Investigation; writing – original draft; writing – review and editing; visualization; methodology. **Bruno Di Geronimo:** Investigation; writing – original draft. **Santiago Alonso‐Gil:** Conceptualization; methodology; investigation; writing – review and editing. **Christoph Grininger:** Investigation; methodology. **Gibu George:** Investigation; writing – original draft. **Ulrika Ferstl:** Investigation; writing – review and editing. **Sereina Annik Herzog:** Investigation. **Bojan Žagrović:** Writing – review and editing. **Christoph Nusshold:** Investigation; writing – review and editing. **Tea Pavkov‐Keller:** Writing – review and editing. **Pedro A. Sánchez‐Murcia:** Conceptualization; investigation; writing – original draft; writing – review and editing; visualization; supervision; validation.

## CONFLICT OF INTEREST STATEMENT

The authors declare that they have no conflicts of interest.

## Supporting information


**Figure S1.** Scheme for the topology of hLAMAN. Image modified from PDBsum.
**Figure S2.** PARENT analysis contact map in hLAMAN.
**Figure S3.** KIF‐WISP calculations from the first‐sphere influence on hLAMAN.
**Figure S4.** KIF‐WISP calculations from the second‐sphere influence on hLAMAN.
**Figure S5.** KIF‐WISP node degeneracy for each path derived from the hLAMAN trajectory.
**Figure S6.** Radar charts for data shown in Table 2.
**Table S1.** Mean ΔΔ*G*_*X*→*Y*_ value (kcal mol^−1^) for the reported missense mutations computed on the MD ensemble of wild‐type hLAMAN.
**Table S2.** Descriptive statistics of the Mann–Whitney *U* test analysis for the ΔΔ*G*_*X*→*Y*_ values (kcal mol^−1^) for the groups folding and activity.
**Table S3.** RMSF (Å) for the reported missense mutations computed along the MD simulation of the hLAMAN monomer.
**Table S4.** Computed bond‐to‐bond propensity scoring and mutual information (MI) for the reported missense mutations in hLAMAN.
**Table S5.** Descriptive statistics of the Mann–Whitney *U* test analysis for the MI values for the groups folding and activity.
**Table S6.** Resides identified on the path from the reported position to the active site using the KIF‐WISP analysis.

## Data Availability

The data that support the findings of this study are openly available in Zenodo at https://zenodo.org/records/11638802.
