# Supplementary material for: A new view of missense mutations in α‐mannosidosis using molecular dynamics conformational ensembles
Source: Protein Sci. 2025 Mar 24;34(4):e70080. doi: 10.1002/pro.70080 (PMC11931667; doi:10.1002/pro.70080)
Supplement: Supplementary file 1 — Figure S1. Scheme for the topology of hLAMAN. Image modified from PDBsum. Figure S2. PARENT analysis contact map in hLAMAN. Figure S3. KIF‐WISP calculations from the first‐sphere influence on hLAMAN. Figure S4. KIF‐WISP calculations from the second‐sphere influence on hLAMAN. Figure S5. KIF‐WISP node degeneracy for each path derived from the hLAMAN trajectory. Figure S6. Radar charts for data shown in Table 2. Table S1. Mean ΔΔG X→Y value (kcal mol−1) for the reported missense mutations computed on the MD ensemble of wild‐type hLAMAN. Table S2. Descriptive statistics of the Mann–Whitney U test analysis for the ΔΔG X→Y values (kcal mol−1) for the groups folding and activity. Table S3. RMSF (Å) for the reported missense mutations computed along the MD simulation of the hLAMAN monomer. Table S4. Computed bond‐to‐bond propensity scoring and mutual information (MI) for the reported missense mutations in hLAMAN. Table S5. Descriptive statistics of the Mann–Whitney U test analysis for the MI values for the groups folding and activity. Table S6. Resides identified on the path from the reported position to the active site using the KIF‐WISP analysis. [file PRO-34-e70080-s001.pdf]

Supporting Information: A new view of missense mutations in α-mannosidosis using molecular dynamics conformational ensembles

Špela Mandl,[†] Bruno Di Geronimo,[†,‡] Santiago Alonso-Gil,[¶,§] Christoph Grininger,[‖] Gibu George,[⊥] Ulrika Ferstl,[†] Sereina Annik Herzog,[#] Bojan Žagrović,[¶,§] Christoph Nusshold,[†] Tea Pavkov-Keller,[‖,@] and Pedro A. Sánchez-Murcia[*,†,@]

†*Laboratory of Computer-Aided Molecular Design, Division of Medicinal Chemistry, Otto-Loewi Research Center, Medical University of Graz, Neue Stiftingtalstr. 6/III, A-8010 Graz, Austria*

‡*Present address: School of Chemistry and Biochemistry, Georgia Institute of Technology, Atlanta, Georgia, USA*

¶*Max Perutz Labs, Vienna Biocenter Campus (VBC), Campus Vienna Biocenter 5, 1030, Vienna, Austria*

§*Department of Structural and Computational Biology, Vienna BioCenter University of Vienna Campus-Vienna-Biocenter 5, A-1030 Vienna, Austria*

‖*Institute of Molecular Biosciences, University of Graz, Humboldtstrasse 50, 8010 Graz, Austria*

⊥*Institut de Química Computacional i Catàlisi and Departament de Química, Universitat de Girona, C/ Maria Aurèlia Capmany 69, 17003 Girona, Catalonia, Spain*

#*Institute for Medical Informatics, Statistics and Documentation, Medical University of Graz, Neue Stiftintalstr. 6/III, A-8010 Graz, Austria*

@*BioTechMed-Graz, Mozartgasse 12/II, A-8010 Graz, Austria*

E-mail: pedro.murcia@medunigraz.at

Supplementary Figures

S1 Scheme for the topology of hLAMAN. Image modified from PDBsum. 5

S2 PARENT analysis contact map in hLAMAN. 6

S3 KIF-WISP calculations from the first-sphere influence on hLAMAN. Source positions are (A) G153, (B) H200, (C) P263, (D) S318, (E) P379, (F) G451 and (G) V457 (depicted as red spheres) and active sites as sinks (depicted as blue spheres). 7

S4 KIF-WISP calculations from the second-sphere influence on hLAMAN. Source positions are (A) G420, (B) T745 (C) G801, and (D) R916 (depicted as red spheres) and active sites as sinks (depicted as blue spheres). 8

S5 Node degeneracy for each path derived from the hLAMAN trajectory are illustrated. Mutant positions are highlighted in red, while active site amino acids are depicted in blue. To enhance clarity, only residues with node degeneracies 0.1 are displayed. 9

S6 Radar charts of the data shown in Table 3. Each of the axes is defined within {0,1}. DDG: $\Delta\Delta G_{X\to Y}$; SD: standard deviation of $\Delta\Delta G_{X\to Y}$; b2b: bond-to-bond propensity scoring. 10

Supplementary Tables

S1 Mean $\Delta\Delta G_{X\to Y}$ value (kcal mol^{-1}) for the reported missense mutations computed on the MD ensemble of wild-type hLAMAN with FoldX. 11

S2 Descriptive statistics of the Mann-Whitney-U test analysis for the $\Delta\Delta G_{X\to Y}$ values (kcal mol^{-1}) for the groups folding and activity. 12

S3 RMSF (Å) values for the positions of the reported missense mutations computed along the MD simulation of the hLAMAN monomer. 13

S4 Computed bond-to-bond propensity scoring and mutual information (MI) for the reported missense mutations in hLAMAN. 14
S5 Descriptive statistics of the Mann-Whitney-U test analysis for the MI values for the groups folding and activity. 15
S6 Resides identified on the path from the reported position to the active site using the KIF-WISP analysis. 15

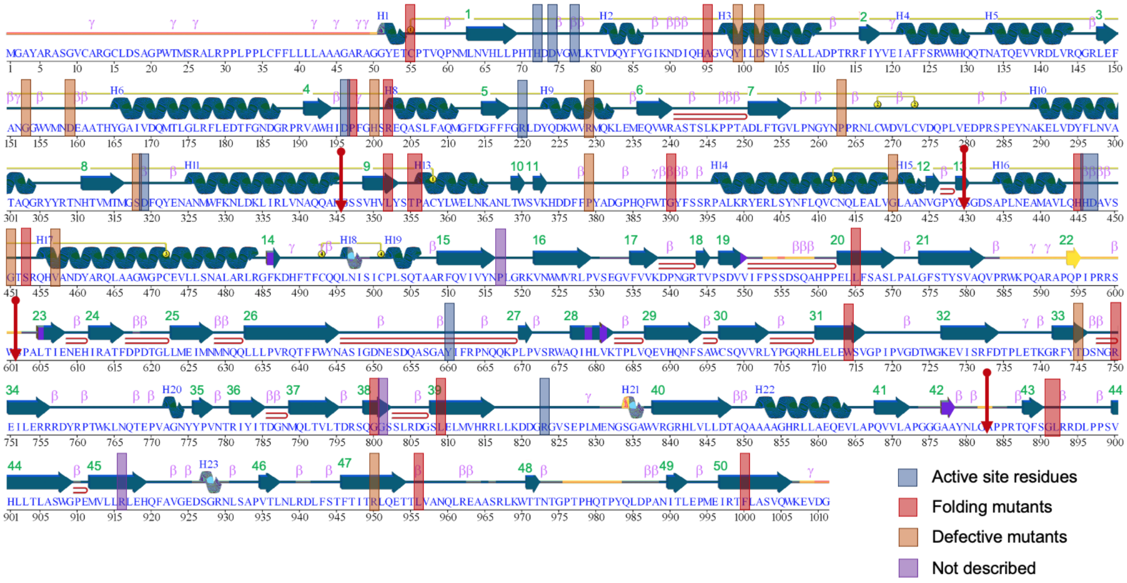

Figure S1: Scheme for the topology of hLAMAN. Image modified from PDBsum.

Figure S2: PARENT analysis contact map in hLAMAN.

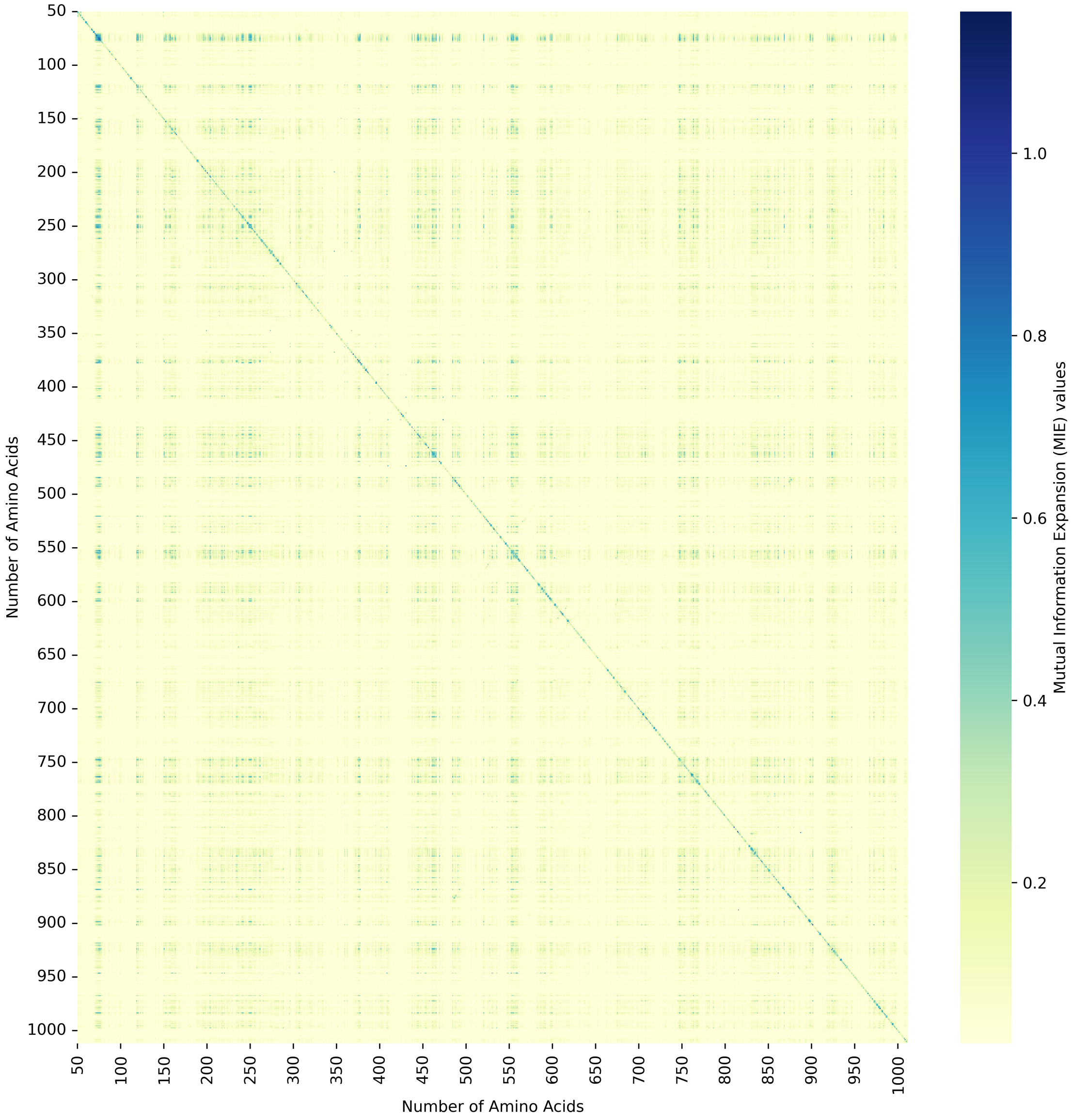

Figure S3: KIF-WISP calculations from the first-sphere influence on hLAMAN. Source positions are (A) G153, (B) H200, (C) P263, (D) S318, (E) P379, (F) G451 and (G) V457 (depicted as red spheres) and active sites as sinks (depicted as blue spheres).

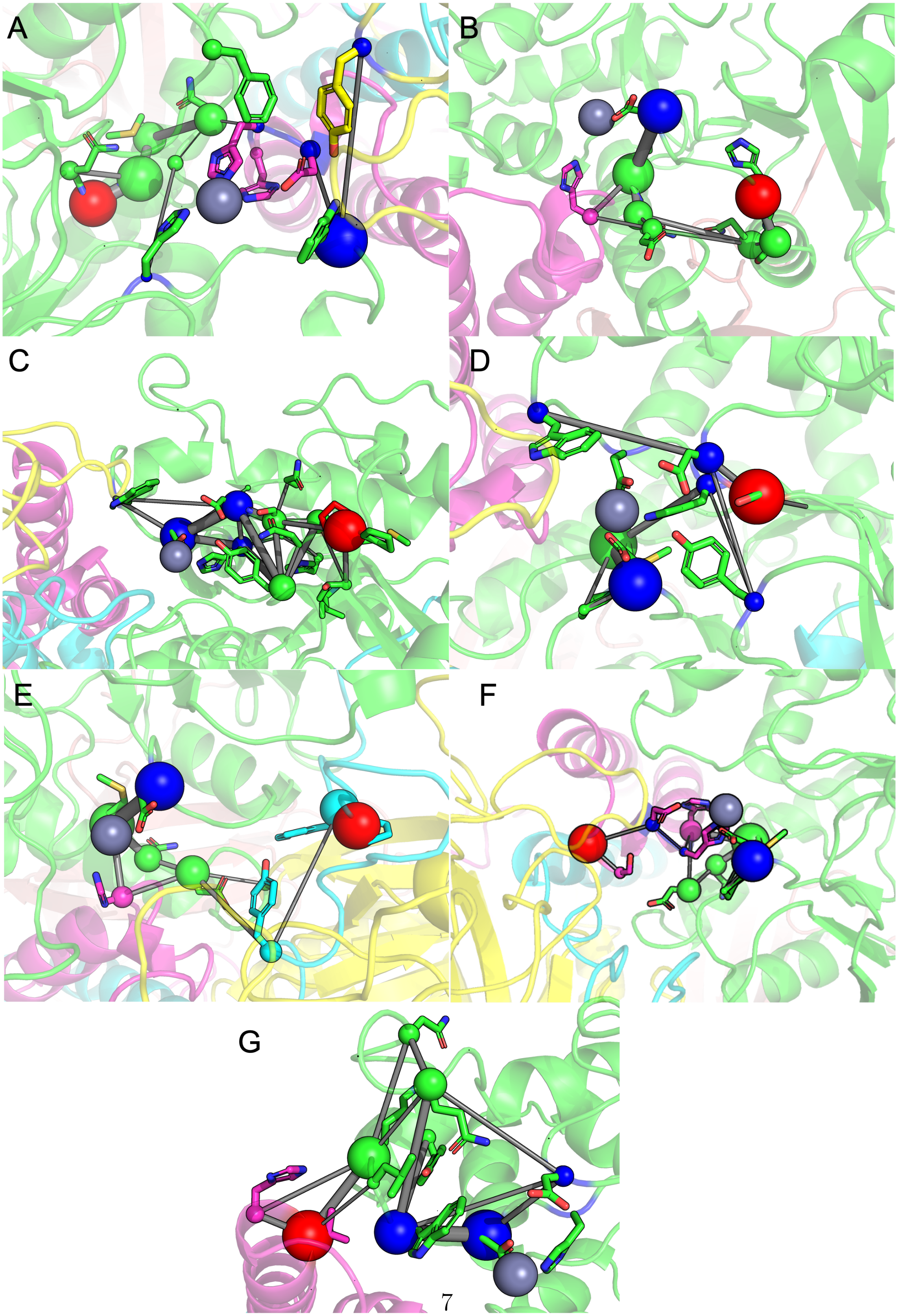

Figure S4: KIF-WISP calculations from the second-sphere influence on hLAMAN. Source positions are (A) G420, (B) T745 (C) G801, and (D) R916 (depicted as red spheres) and active sites as sinks (depicted as blue spheres).

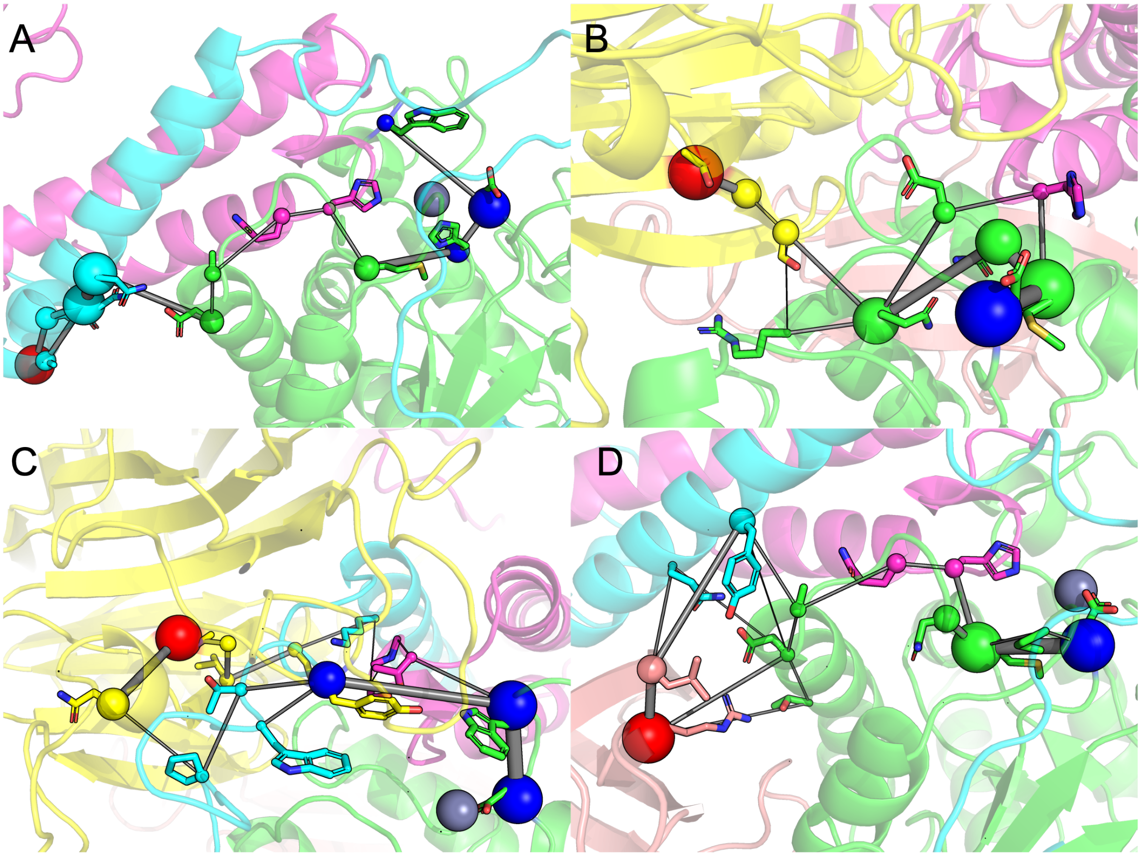

Figure S5: Node degeneracy for each path derived from the hLAMAN trajectory are illustrated. Mutant positions are highlighted in red, while active site amino acids are depicted in blue. To enhance clarity, only residues with node degeneracies 0.1 are displayed.

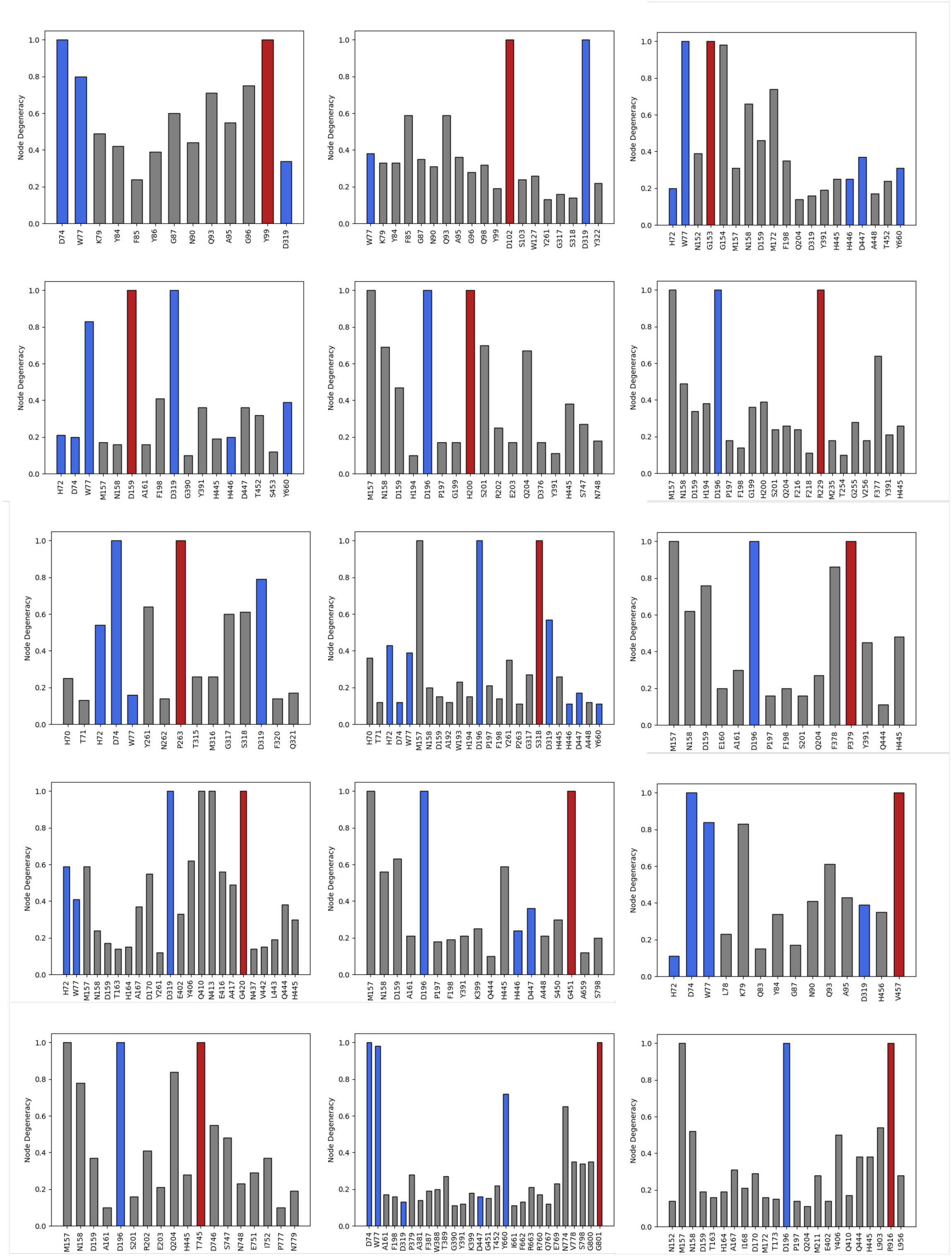

Figure S6: Radar charts of the data shown in Table 3. Each of the axes is defined within $\{0,1\}$. DDG: $\Delta\Delta G_{X\rightarrow Y}$; SD: standard deviation of $\Delta\Delta G_{X\rightarrow Y}$; b2b: bond-to-bond propensity scoring.

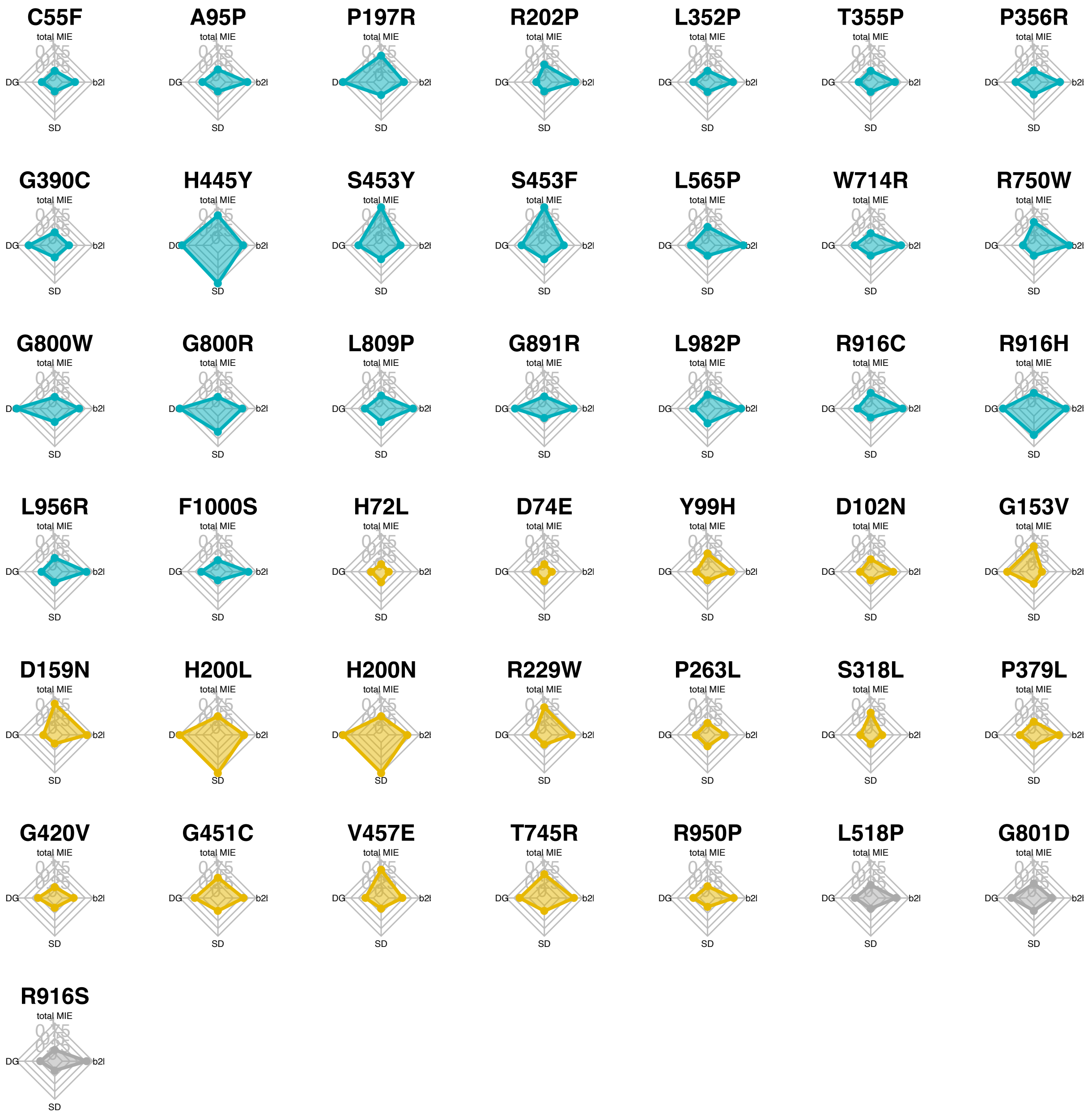

Table S1: Mean $\Delta\Delta G_{X \to Y}$ value (kcal mol^{-1}) for the reported missense mutations computed on the MD ensemble of wild-type hLAMAN with FoldX.

Mutant	$\Delta\Delta G_{X \to Y}$ (kcal mol^{-1})	SD($\Delta\Delta G_{X \to Y}$) (kcal mol^{-1})
**(a) Folding**		
C55F	3.50	1.06
A95P	5.24	1.10
P197R	20.35	3.37
R202P	-0.22	1.12
L352P	4.26	1.24
T355P	2.87	1.34
P356R	6.98	2.87
G390C	12.27	2.74
H445Y	18.91	98.57
S453Y	14.25	3.87
S453F	10.07	3.87
L565P	5.99	1.64
W714R	5.49	1.61
R750W	2.15	3.63
G800W	42.81	9.74
G800R	14.89	3.52
L809P	5.80	1.25
G891R	14.52	4.39
L892P	4.48	1.41
R916C	3.70	0.98
R916H	15.55	11.73
L956R	3.68	1.60
F1000S	6.01	0.75
**(b) Defective activity**		
H72L	-	-
D74E	-	-
Y99H	2.37	0.73
D102N	1.86	0.71
G153V	12.67	2.97
D159N	2.48	0.81
H200L	203.91	339.97
H200N	203.76	339.92
R229W	1.88	1.36
P263L	2.68	2.27
S318L	1.72	1.08
P379L	4.23	1.81
G420V	6.12	1.31
G451C	10.45	3.31
V457E	5.42	1.73
T745R	11.38	3.39
R950P	4.33	0.81
**(c) Non-classified**		
L518P	5.53	1.81
G801D	9.87	3.18
R916S	4.61	1.09

N.D. no data. *Distance to the Zn^{2+} atom of the active site. **Average value from the analysis of 9 MD snapshots. ***Average value from 10 000 MD snapshots.

Table S2: Descriptive statistics of the Mann-Whitney-U test analysis for the $\Delta\Delta G_{X \to Y}$ values (kcal mol^{-1}) for the groups folding and activity.

**Characteristic**	**folding, N = 23**	**activity, N = 17**
Median (IQR)	0.30 (0.21 - 0.74)	0.21 (0.09 - 0.51)
Range	0.01 - 1.00	0.05 - 1.00

Table S3: RMSF (Å) values for the positions of the reported missense mutations computed along the MD simulation of the hLAMAN monomer.

Mutant	RMSF (Å)
**(a) Folding**	
C55	0.652
A95	0.696
P197	0.467
R202	0.518
L352	0.550
T355	0.497
P356	0.514
G390	0.428
H445	0.407
S453	0.487
S453	0.487
L565	0.579
W714	0.527
R750	0.468
G800	0.444
G800	0.444
L809	0.470
G891	0.564
L892	0.610
R916	0.429
R916	0.429
L956	0.478
F1000	0.499
**(b) Defective activity**	
H72	0.556
D74	0.628
Y99	0.676
D102	0.643
G153	0.513
D159	0.371
H200	0.482
H200	0.482
R229	1.151
P263	1.105
S318	0.684
P379	0.508
G420	0.649
G451	0.519
V457	0.456
T745	0.462
R950	0.768
**(c) Non-classified**	
L518	0.442
G801	0.456
R916	0.429

Table S4: Computed bond-to-bond propensity scoring and mutual information (MI) for the reported missense mutations in hLAMAN.

Mutant	Distance* (Å)	bond-to-bond scoring**	Total MI value***
**(a) Folding**			
C55F	27.3	0.14	0.00
A95P	13	0.58	0.16
P197R	7.0	0.51	0.29
R202P	20.0	0.79	0.14
L352P	11.0	0.54	0.26
T355P	19.4	0.51	0.26
P356R	17.9	0.71	0.24
G390C	17.1	0.15	0.00
H445Y	8.2	0.70	0.52
S453Y	10.2	0.70	0.43
S453F	10.2	0.70	0.43
L565P	45.1	0.89	0.15
W714R	32.1	0.75	0.11
R750W	24.7	0.93	0.20
G800W	20.8	0.63	0.18
G800R	20.8	0.63	0.18
L809P	31.7	0.74	0.40
G891R	40.7	0.80	0.16
L892P	41.8	0.88	0.16
R916C	29.8	0.79	0.39
R916H	29.8	0.79	0.39
L956R	24.8	0.85	0.16
F1000S	32.4	0.74	0.32
**(b) Defective activity**			
H72L	6.5	0.00	0.95
D74E	5.0	0.00	0.86
Y99H	18.5	0.57	0.17
D102N	21.0	0.61	0.13
G153V	14.9	0.02	0.28
D159N	9.7	0.82	0.39
H200L	13.9	0.66	0.17
H200N	13.9	0.66	0.17
R229W	23.7	0.65	0.33
P263L	15.4	0.37	0.12
S318L	9.3	0.3	0.24
P379L	18.1	0.51	0.64
G420V	37.6	0.42	N.D.
G451C	12.9	0.70	0.16
V457E	14.0	0.61	0.17
L518P	34.5	0.58	0.08
T745R	25.5	0.70	0.29
**(c) Non-classified missense mutations**			
G801D	24.3	0.42	0.18
R916S	29.8	0.79	0.39
R950P	41.2	0.58	0.16

N.D. no data. *Distance to the Zn^{2+} atom of the active site. **Average value from the analysis of 9 MD snapshots. ***Average value from 10,000 MD snapshots.

Table S5: Descriptive statistics of the Mann-Whitney-U test analysis for the MI values for the groups folding and activity.

**Characteristic**	**folding, N = 23**	**activity, N = 17**
Median (IQR)	0.18 (0.13 - 0.34)	0.36 (0.17 - 0.56)
Range	0.12 - 1.00	0.11 - 0.77
Missing	0	2

Table S6: Resides identified on the path from the reported position to the active site using the KIF-WISP analysis.

**Position**	**KIF sub-optimal pathway**
99	99, 96, 86, 85, 84, 79, 77*, 95, 90, 93, 319*
102	102, 99, 98, 96, 9, 319*, 93, 87, 85, 84, 87, 79, 77*
153	153, 154, 172, 158, 198, 446*, 445, 447*, 72*, 77*, 660*
159	159, 198, 660*, 445, 446*, 447*, 72*, 319*, 77*
200	200, 201, 204, 158, 159, 157, 445, 196*
229	229, 377, 200, 204, 104, 197, 158, 157, 319*
263	263, 316, 262, 261, 315, 317, 318, 319*, 320, 321, 70, 71, 72*, 74*, 77*
318	318, 319*, 272, 261, 196*, 197, 74*, 77*
379	379, 378, 391, 159, 158, 157, 445, 196*
420	420, 416, 417, 413, 410, 170, 167, 444, 157, 445, 72*, 319*, 77*
451	451, 450, 446*, 445, 159, 158, 157, 197, 196*
457	457, 456 79, 79 77*, 93, 74*, 72*, 319*
518	not found
745	745, 746, 747, 202, 204, 158, 159, 157, 196*, 445*
801	800, 801, 778, 798, 399, 450, 451, 452, 74*, 77*, 774, 379, 389, 388, 660*
916	916, 903, 410, 406, 170, 173, 167, 444, 445, 158, 157, 197, 196*
950	not found

*Residue located at the active site.
